# The deacetylase SIRT6 promotes the repair of UV-induced DNA damage by targeting DDB2

**DOI:** 10.1093/nar/gkaa661

**Published:** 2020-08-13

**Authors:** Anke Geng, Huanyin Tang, Jin Huang, Zhen Qian, Nan Qin, Yunxia Yao, Zhu Xu, Hao Chen, Li Lan, Hongjuan Xie, Jian Zhang, Ying Jiang, Zhiyong Mao

**Affiliations:** Clinical and Translational Research Center of Shanghai First Maternity & Infant Hospital, Shanghai Key Laboratory of Signaling and Disease Research, Frontier Science Center for Stem Cell Research, School of Life Sciences and Technology, Tongji University, Shanghai 200092, China; Clinical and Translational Research Center of Shanghai First Maternity & Infant Hospital, Shanghai Key Laboratory of Signaling and Disease Research, Frontier Science Center for Stem Cell Research, School of Life Sciences and Technology, Tongji University, Shanghai 200092, China; Key Laboratory of Environmental Pollution Monitoring and Disease Control of Ministry of Education, Guizhou Medical University, Guiyang 550025, China; Clinical and Translational Research Center of Shanghai First Maternity & Infant Hospital, Shanghai Key Laboratory of Signaling and Disease Research, Frontier Science Center for Stem Cell Research, School of Life Sciences and Technology, Tongji University, Shanghai 200092, China; Clinical and Translational Research Center of Shanghai First Maternity & Infant Hospital, Shanghai Key Laboratory of Signaling and Disease Research, Frontier Science Center for Stem Cell Research, School of Life Sciences and Technology, Tongji University, Shanghai 200092, China; Clinical and Translational Research Center of Shanghai First Maternity & Infant Hospital, Shanghai Key Laboratory of Signaling and Disease Research, Frontier Science Center for Stem Cell Research, School of Life Sciences and Technology, Tongji University, Shanghai 200092, China; College of Pharmacy, Yanbian University, Yanji 133002, Jilin Province, China; Clinical and Translational Research Center of Shanghai First Maternity & Infant Hospital, Shanghai Key Laboratory of Signaling and Disease Research, Frontier Science Center for Stem Cell Research, School of Life Sciences and Technology, Tongji University, Shanghai 200092, China; University of Pittsburgh Cancer Institute, University of Pittsburgh School of Medicine, Pittsburgh, PA 15213, USA; University of Pittsburgh Cancer Institute, University of Pittsburgh School of Medicine, Pittsburgh, PA 15213, USA; Clinical and Translational Research Center of Shanghai First Maternity & Infant Hospital, Shanghai Key Laboratory of Signaling and Disease Research, Frontier Science Center for Stem Cell Research, School of Life Sciences and Technology, Tongji University, Shanghai 200092, China; Department of Pathophysiology, Key Laboratory of Cell Differentiation and Apoptosis of Ministry of Education, Shanghai Jiao-Tong University School of Medicine, 200025 Shanghai, China; Clinical and Translational Research Center of Shanghai First Maternity & Infant Hospital, Shanghai Key Laboratory of Signaling and Disease Research, Frontier Science Center for Stem Cell Research, School of Life Sciences and Technology, Tongji University, Shanghai 200092, China; Clinical and Translational Research Center of Shanghai First Maternity & Infant Hospital, Shanghai Key Laboratory of Signaling and Disease Research, Frontier Science Center for Stem Cell Research, School of Life Sciences and Technology, Tongji University, Shanghai 200092, China; Tsingtao Advanced Research Institute, Tongji University, 67 Yinchuan West Road, Qingdao 266071, China

## Abstract

The NAD+-dependent deacetylase and mono-ADP-ribosyl transferase SIRT6 stabilizes the genome by promoting DNA double strand break repair, thereby acting as a tumor suppressor. However, whether SIRT6 regulates nucleotide excision repair (NER) remains unknown. Here, we showed that SIRT6 was recruited to sites of UV-induced DNA damage and stimulated the repair of UV-induced DNA damage. Mechanistic studies further indicated that SIRT6 interacted with DDB2, the major sensor initiating global genome NER (GG-NER), and that the interaction was enhanced upon UV irradiation. SIRT6 deacetylated DDB2 at two lysine residues, K35 and K77, upon UV stress and then promoted DDB2 ubiquitination and segregation from chromatin, thereby facilitating downstream signaling. In addition, we characterized several SIRT6 mutations derived from melanoma patients. These SIRT6 mutants ablated the stimulatory effect of SIRT6 on NER and destabilized the genome due to (i) partial loss of enzymatic activity (P27S or H50Y), (ii) a nonsense mutation (R150*) or (iii) high turnover rates (G134W). Overall, we demonstrate that SIRT6 promotes NER by deacetylating DDB2, thereby preventing the onset of melanomagenesis.

## INTRODUCTION

The nucleotide excision repair (NER) pathway is responsible for the removal of bulky DNA adducts induced by UV irradiation or chemicals, such as cisplatin (1). NER can be further divided into two subpathways, global genome NER (GG-NER) and transcription-coupled NER (TC-NER) (2,3). GG-NER can occur in any region of the genome, while TC-NER repairs DNA damage only at transcriptionally active loci. The two subpathways differ in the DNA damage recognition steps. During the GG-NER process, DNA lesions are recognized by XPC-RAD23B/RAD23A and UV-DDB. For TC-NER, RNA polymerases stalled by bulky DNA adducts, together with CSA and CSB, initiate the repair process. After the recognition step, the two subpathways share identical repair machinery, composed of factors including TFIIH, RPA, XPA, XPB, XPD, ERCC1, XPF and XPG, which cooperate to complete the repair process. Defects in the NER pathway lead to a number of human syndromes, including xeroderma pigmentosum (XP), Cockayne syndrome (CS) and trichothiodystrophy (TTD) (4). Patients deficient in any of several NER genes are extremely sensitive to UV irradiation and prone to skin tumorigenesis. Among all types of skin cancers, melanoma is very rare and accounts for ∼4% of dermatological cancers, but it is the leading cause of skin cancer-related death (5). Interestingly, Cockayne syndrome, which is caused by TC-NER defects, is mainly characterized by neurological or developmental disorders but not a heightened incidence of melanoma (4). However, patients with xeroderma pigmentosum, which results from dysfunctional GG-NER, have an approximately 1000-fold increase in melanoma incidence in comparison to control individuals (6).

SIRT6, one of the seven mammalian homologs of yeast Sir2, is well known for its roles in regulating tumorigenesis and longevity (7). Increased genomic instability is a common hallmark of both cancer and aging (8,9). Defects in different types of DNA repair contribute to rising genomic instability, leading to tumorigenesis or the onset of aging (10–12). SIRT6 maintains genome integrity by promoting DNA repair through different means. Great progress has been made in understanding the role of SIRT6 in regulating homologous recombination (HR) and nonhomologous end joining (NHEJ), two major DNA double strand break (DSB) repair pathways. As a mono-ADP-ribosyl transferase, SIRT6 adds an ADP-ribose to PARP1 at residue K521, thereby promoting both HR and alternative NHEJ under oxidative stress (13). Independent of its enzymatic activity, SIRT6 facilitates the recruitment of the chromatin remodeler SNF2H to DNA damage sites, resulting in relaxation of the local chromatin structure and promotion of HR-directed repair (14). Our group has previously demonstrated that in induced pluripotent stem cells, mouse SIRT6 physically binds to Ku80 and facilitates the phosphorylation of DNA-PKcs in response to DNA damage (15), eventually leading to efficient canonical NHEJ. In addition to its role in regulating DSB repair, SIRT6 was originally identified as a factor regulating base excision repair (BER) (16). Loss of SIRT6 in mice sensitizes mouse cells to oxidative stress (16). Our group has demonstrated that similar to its regulation of DSB repair, SIRT6 promotes BER in a PARP1-dependent manner (17). Surprisingly, whether SIRT6 participates in NER and the potential associated regulatory mechanisms remain largely undetermined.

Here, we demonstrate that SIRT6 promotes DNA repair through GG-NER by targeting DDB2. SIRT6 is recruited to sites of UV-induced DNA damage and interacts with DDB2 upon stress. In response to UV irradiation, SIRT6 deacetylates DDB2 at two lysine residues, K35 and K77, thereby promoting the ubiquitination of DDB2 and segregation of DDB2 from chromatin, eventually facilitating NER signal transduction. Moreover, through data mining, we identified 8 mutations in SIRT6 in melanoma patients. Functional analysis revealed that four of these SIRT6 mutants reduced the ability to preserve genome integrity through NER due to loss of enzymatic activity, truncation of the functional protein or induction of high turnover rates, thereby increasing the chance of acquiring a high burden of mutations and eventually promoting melanomagenesis.

## MATERIALS AND METHODS

### Cell culture

All HCA2-hTERT fibroblasts were maintained in MEM (HyClone, Logan, UT, USA, Cat. #SH30234) supplemented with 10% FBS (Gibco, Carlsbad, CA, USA, Cat. # 16000), 1% penicillin/streptomycin (HyClone, Cat. #SV30010) and 1% NEAA (HyClone, Cat. # SH3023801). HEK293, U2OS and human melanoma A875 cells were grown in DMEM (Sigma, Cat. # D6429) supplemented with 10% FBS and 1% penicillin/streptomycin. All cells were cultured in a 37°C incubator with 5% CO_2_.

### Plasmids, siRNA, shRNAs and antibodies

The ORF of DDB2 was amplified from cDNA derived from 293 cells and subsequently cloned into the pEGFP-N1 backbone by replacing the EGFP ORF using the restriction enzymes SalI and NotI. The information for other vectors encoding SIRT6 WT and SIRT6 mutants is as described previously (13).

The sequences of siRNAs against SIRT6 used in HCA2-hTERT cells were as follows: siSIRT6-1: 5′-AAGCUGGAGCCCAAGGAGGTT-3′; and siSIRT6-2: 5′-CCCCCUACAGCCCACCCUATT-3′. The sequences of siRNAs against SIRT6 used in the A875 cell line were as follows: siSIRT6-1: 5′- CCCCCUACAGCCCACCCUATT-3′; and siSIRT6-2: 5′-TCATGACCCGGCTCATGAATT-3′. The sequences of siRNAs against DDB2 were as follows: siDDB2-1, 5′-GAGCGAGAUCCGUGUUUAC-3′; and siDDB2-2, 5′-UCUCTGGGCUGUUGUUUAA-3′. The sequences of siRNAs against PKM2 were as follows: siPKM2-1, 5′-CCAGATGGCAAGAGGGTGA-3′; and siPKM2-2, 5′-CAUCUACCACUUGCAAUUATT-3′. The sequences of siRNAs against CSA were as follows: siCSA-1, 5′-TGATGATGAGACTACAACAAA-3′; and siCSA-2, 5′-GCGCTAATGCTTGAACTCTTT-3′.

The pLKO1 vector was utilized as the backbone for cloning shRNA viral vectors. The sequences targeting SIRT6 used in HCA2-hTERT cells were as follows: shSIRT6-1: 5′-GCCTCTGACTTGCTGTGTTGT-3′; and shSIRT6-2: 5′-AAGAATGTGCCAAGTGTAAGA-3′. The sequences targeting SIRT6 used in the A875 cell line were as follows: shSIRT6-1: 5′-CAGTACGTCCGAGACACAGTC-3′; and shSIRT6-2: 5′-CAAGTTCGACACCACCTTTGA-3′. The sequences targeting XPC were as follows: shXPC-1: 5′-CCCACTGCCATTGGCTTATAT-3′; and shXPC-2: 5′-GCAAATGGCTTCTATCGAATT-3′.

The antibodies used in this study were as follows: anti-SIRT6 (Abcam, Cat. #62738; Abcam, Cat. #62739), anti-β-TUBULIN (CMCTAG, Cat. # AT0050), anti-DDB2 (Cell Signaling Technology, Cat. # 5416), anti-XPC (Cell Signaling Technology, Cat. # 14768), anti-HA (Cell Signaling Technology, Cat. # 3724), anti-His (Abcam, Cat. # 9108), anti-GFP (Abcam, Cat. # ab290), anti-K48-linked specific polyubiquitin (Cell Signaling Technology, Cat. # 8081), anti-Flag (Abclonal, Cat. # AE005), anti-acetyl lysine (Cell Signaling Technology, Cat. # 9441), and anti-GFP-Trap (Chromotek, Cat. # gta-10).

### Transfections

Fibroblasts and human melanoma A875 cells were transfected with the indicated plasmids or siRNAs on a Lonza 4D machine (DT-130 program). For HEK293 cells, exogenous DNA was introduced into cells using polyethylenimine (PEI) transfection (18).

### FACS analysis

On day 3 post transfection, cells were harvested and resuspended in 200 μl 1× PBS for FACS analysis on a FACS Verse (BD Biosciences, USA). At least 20 000 events were counted. All results were analyzed using FlowJo software. For FACS analysis, all experiments were repeated at least three times, and a two-sided *t*-test was used to calculate *P* values.

### Apoptosis assay

Cells (1 × 10^5^) were seeded in 6-well plates and cultured for 24 h before being irradiated with UVC rays at 20 J/m^2^. At 72 h after irradiation, the cells were harvested for annexin V staining using the FITC Annexin V Apoptosis Detection Kit I (BD Pharmingen).

### Immunoprecipitation

Harvested cells were lysed with a lysis buffer (20 mM HEPES pH 8.0, 0.2 mM EDTA, 5% glycerol, 150 mM NaCl, and 1% NP-40) on ice for 15 min. The lysate was then sonicated on ice at 10% power for 2 min. After centrifuging at 13 500 rpm for 15 min at 4°C, the supernatants were precleared by incubation with 20 μl protein A/G agarose (Abmart, #A10001M) and IgG (Santa Cruz, #sc-2025) for 1 h at 4°C. The samples were then centrifuged at 1000 rpm for 3 min at 4°C. The supernatants were incubated with antibodies overnight. Then, protein A/G agarose was added to the supernatants and incubated for 2 h at 4°C. The precipitates were washed three times with the lysis buffer, boiled for 10 min with 2× SDS sample buffer and resolved by SDS-PAGE.

For the precipitation of ubiquitinated DDB2-FLAG under denaturing conditions, cells were lysed in SDS lysis buffer (50 mM Tris–HCl pH 6.8, 5 mM DTT and 1% (w/v) SDS), followed by sonication on ice at 10% power for 4 min. After centrifuging at 13 500 rpm for 15 min at 4°C, the supernatant was washed four times with NP-40 buffer (25 mM Tris–HCl pH 8.0, 0.3 mM NaCl, 1 mM EDTA, 10% (v/v) glycerol, 1% (v/v) NP-40 and EDTA-free protease inhibitor cocktail) and incubated with 20 μl protein A/G agarose (Abmart, #A10001M) or IgG (Santa Cruz, #sc-2025) for 1 h at 4°C, followed by performance of the procedures described above.

### Laser microirradiation

Laser microirradiation experiments were performed with a Leica DM6500 confocal microscopy 405-nm laser diode system. In the experiments, U2OS cells were kept at 37°C in the Oko-Touch (Okolab) controlled heated chamber. A high-throughput microscope equipped with an ultraviolet light-transmitting HC PL APO CS2 63×/1.40 oil objective was used to visualize living cells. In the absence or presence of pretreatment with 100 μM 8-methoxypsoralen (8-mop) for 2–5 min, U2OS cells were microirradiated with a 50-mW, 405-nm laser diode and a FRAP model was employed. Laser light was passed through a 63× oil immersion objective lens. Images were acquired using Leica LAS AF software (LAS AF3.1.0) and further analyzed using LAS X software.

### LC–MS/MS analysis and data processing

Protein samples prepared for mass spectrometry were separated by SDS-PAGE. After Coomassie blue staining, the gel strip containing the protein of interest was cut out and sent to PTM Biolabs for analysis (Hangzhou, Zhejiang, China). The detailed experimental methods are provided in the Supplementary Information.

### Chromatin isolation

Cells were resuspended in 200 μl buffer A (10 mM HEPES pH 7.9, 10 mM KCl, 1.5 mM MgCl_2_, 0.34 M sucrose, 10% glycerol, 1 mM DTT and protease inhibitors), followed by treatment with 0.1% Triton X-100 and incubation on ice for 10 min. The cytoplasmic extract was separated by low-speed centrifugation (4 min at 1300 g, 4°C). The pelleted nuclei were washed once with buffer A and then lysed for 30 min on ice in 200 μl buffer B (3 mM EDTA, 0.2 mM EGTA, 1 mM DTT and protease inhibitors). Insoluble chromatin was pelleted by centrifugation (4 min at 1700 g, 4°C), washed once with buffer B and separated by centrifugation at high speed (10 000 g) for 1 min. The chromatin pellet was resuspended in 50 μl buffer A containing 1 mM CaCl_2_ and 50 units MNase and incubated at 37°C for 20 min, followed by the addition of 1 mM EGTA to stop nuclease digestion. Then, 50 μl 2× SDS sample buffer was added to the lysates and boiled for 10 min for further western blot analysis.

### Computational analysis

SIRT6 somatic mutations were identified in TCGA, ICGC and COSMIC skin melanoma samples. Variations called from whole-genome and whole-exome sequencing of TCGA and ICGC skin melanoma patients were used to calculate the nonsynonymous mutation number. Significance was calculated using a two-tailed *t*-test.

## RESULTS

### SIRT6 promotes the repair of UV-induced DNA damage

**Figure 1.**
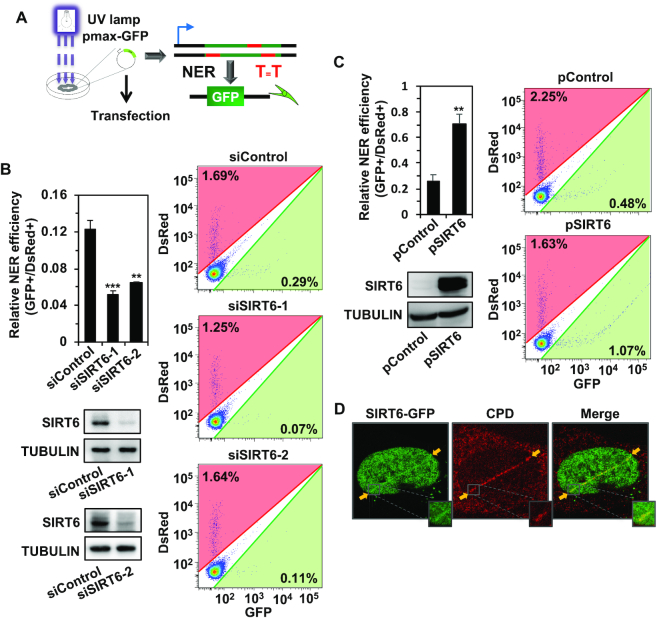
SIRT6 promotes the repair of UV-induced DNA damage. (**A**) Diagram of the plasmid reactivation assay for measuring NER efficiency. Fifty microliters of pmax-GFP (0.01 μg/μl) was treated with UVC irradiation. Then, the damaged pmax-GFP was transfected into cells for further FACS analysis. (**B**) Effect of SIRT6 depletion on NER. Control and SIRT6-depleted fibroblasts were transfected with 0.06 μg UVC-treated pmax-GFP together with 15 ng pDsRed2-N1 as an internal control for normalizing transfection efficiency. On day 3 post transfection, cells were harvested for FACS analysis. The ratio of GFP+ cells to DsRed+ cells was employed as the measure of NER efficiency. Western blot analysis of SIRT6 in control and SIRT6-knockdown cells was performed. All experiments were repeated at least three times. Error bars represent the s.d. ***P < 0.001, **P < 0.01. (**C**) Effect of SIRT6 overexpression on NER. HCA2-hTERT fibroblasts were transfected with 0.06 μg UVC-treated pmax-GFP together with 15 ng pDsRed2-N1 and 5 μg control vector or vector encoding SIRT6, followed by FACS analysis on day 3 post transfection. Western blot analysis of SIRT6 overexpression in fibroblasts was performed. All experiments were repeated at least three times. Error bars represent the s.d. **P < 0.01. (**D**) Representative immunofluorescence images of the recruitment of GFP-SIRT6 and CPD to DNA damage sites in U2OS cells following laser microirradiation in the absence of a photosensitizer. U2OS cells were transfected with 1 μg GFP-SIRT6 plasmid, and at 24 h post transfection, the cells were irradiated with a laser and immunostained with anti-CPD and anti-GFP antibodies.

To examine whether SIRT6 participates in the NER pathway, we employed a plasmid reactivation assay using pmax-GFP plasmids to measure NER efficiency (19). Purified pmax-GFP plasmids were irradiated with increasing doses of UVC light (Figure 1A, Supplementary Figure S1A) and then transfected into HCA2-hTERT cells, an immortalized human fibroblast cell line, followed by FACS analysis of harvested cells at 72 h post transfection. In support of the robustness of this assay, we observed that increasing doses of UV irradiation resulted in lower levels of GFP expression (Supplementary Figure S1A). We then introduced 1200 J/m^2^ UVC-irradiated pmax-GFP together with pDsRed2-N1, which was used to normalize for differences in transfection efficiencies, into fibroblasts. The ratio of GFP+ cells to DsRed+ cells was employed as the measure of NER efficiency. We further validated this assay in XPC-depleted or CSA-depleted HCA2-hTERT cells and observed a significant reduction in NER efficiency in the cells with XPC or CSA expression knocked down (Supplementary Figure S1B, C).

We then tested NER efficiency in SIRT6-depleted cells and SIRT6-overexpressing cells. We found that in HCA2-hTERT cells with SIRT6 depleted using siRNA against SIRT6, NER efficiency was significantly reduced (Figure 1B). Consistently, we observed the same reduction in NER efficiency in SIRT6-knockout MEFs in comparison to wild-type MEFs (Supplementary Figure S2A, B). In addition, depleting SIRT6 using siRNA in A875 cells, a melanoma cell line, also led to a significant reduction in NER (Supplementary Figure S2C, D). Moreover, SIRT6 overexpression significantly enhanced the efficiency of NER in both HCA2-hTERT cells (Figure 1C) and A875 cells (Supplementary Figure S2E, F). Because SIRT6 overexpression augments the efficiency of DSB repair, we reasoned that the stimulation we observed in the NER plasmid reactivation assay might be a consequence of linearized pmax-GFP caused by UVC irradiation. To rule out this possibility, we performed gel electrophoresis experiments with untreated plasmid, plasmid linearized with a restriction enzyme, and UVC-irradiated plasmid samples. We observed that UVC treatment did not result in linearized pmax-GFP plasmids (Supplementary Figure S1D), strongly indicating that the change in NER efficiency related to the presence or absence of SIRT6 was not an artifact resulting from an alteration in the DSB repair capacity. Overall, we identified SIRT6 as a critical factor in the regulation of the process of UV-induced DNA damage repair.

To examine whether SIRT6 exerts its NER function at DNA damage sites, we performed UV laser microirradiation experiments in U2OS cells in the absence or presence of a photosensitizer such as 8-mop to facilitate the generation of DSBs by laser microirradiation (20). We confirmed that the removal of 8-mop abrogated the recruitment of Ku70, which is a critical canonical NHEJ factor, to sites of laser-induced DNA damage (Supplementary Figure S3A, B). Using this assay, we demonstrated that SIRT6 was recruited to sites of UV-induced DNA damage and colocalized with cyclobutane pyrimidine dimers (CPD) (Figure 1D, Supplementary Figure S3C), which are the major type of UV-induced DNA damage. These data indicate that SIRT6 participates in NER at sites of UV-induced DNA damage.

**Figure 2.**
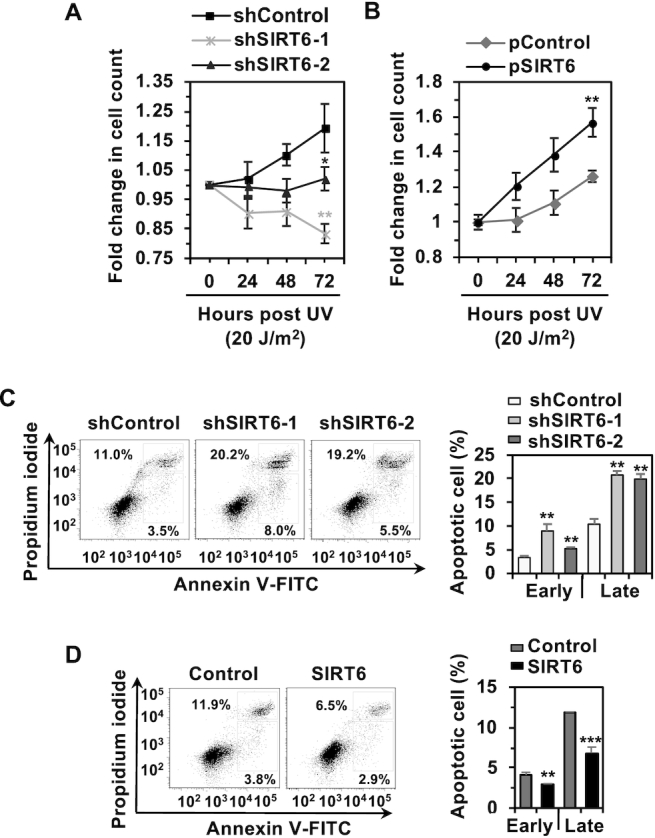
SIRT6 promotes cell survival by suppressing UV-induced apoptosis. (**A**) Depleting SIRT6 inhibits cell proliferation in response to UVC irradiation. HCA2-hTERT cells were exposed to 20 J/m^2^ UVC, followed by cell counting at 24, 48 and 72 h post UV irradiation. Fold changes were calculated as the cell number at the indicated time point post UV irradiation versus the starting number of cells before irradiation. Error bars represent the s.d. **P < 0.01, *P < 0.05. (**B**) SIRT6 overexpression promotes cell proliferation upon UV irradiation. The numbers of control and SIRT6-overexpressing cells were analyzed as described in (A). Error bars represent the s.d. **P < 0.01. (**C**) SIRT6 depletion significantly stimulates UVC-induced apoptosis. On day 3 post UVC irradiation at a dose of 20 J/m^2^, control or SIRT6-depleted HCA2-hTERT cells were harvested for analysis of apoptosis rates. Error bars represent the s.d. **P < 0.01. (**D**) SIRT6 overexpression significantly suppresses UVC-induced apoptosis. On day 3 post UVC irradiation at a dose of 20 J/m^2^, control or SIRT6-overexpressing HCA2-hTERT cells were harvested for analysis of apoptosis rates. In the representative FACS traces shown in (C) and (D), the dots in the upper right rectangle represent cells in the late stage of apoptosis, while those in the lower right rectangle represent cells in the early stage of apoptosis. Error bars represent the s.d. ***P < 0.001, **P < 0.01.

To understand the physiological role of SIRT6 in the context of cell survival, we examined changes in cell proliferation and the apoptosis rate in response to UV irradiation related to the presence or absence of SIRT6. Intriguingly, we found that, in contrast to a previous report (16), loss of SIRT6 significantly impaired cell proliferation after UV irradiation in both HCA2-hTERT cells and A875 cells (Figure 2A, Supplementary Figure S4), while SIRT6 overexpression significantly stimulated cell proliferation in response to UV irradiation (Figure 2B). To further examine whether the observed change in cell proliferation was a consequence of changes in apoptosis, we performed Annexin V/PI staining to analyze apoptosis rates. We found that SIRT6 depletion significantly increased the rates of UV-induced early and late apoptosis on day 3 post UV irradiation (Figure 2C), while SIRT6 overexpression significantly suppressed the rates of UV-induced apoptosis (Figure 2D).

Altogether, these data strongly suggest a key role for SIRT6 in maintaining genome integrity by promoting NER and preventing UV-induced apoptosis.

### The repair of UV-induced DNA damage by SIRT6 is dependent on its enzymatic activities

**Figure 3.**
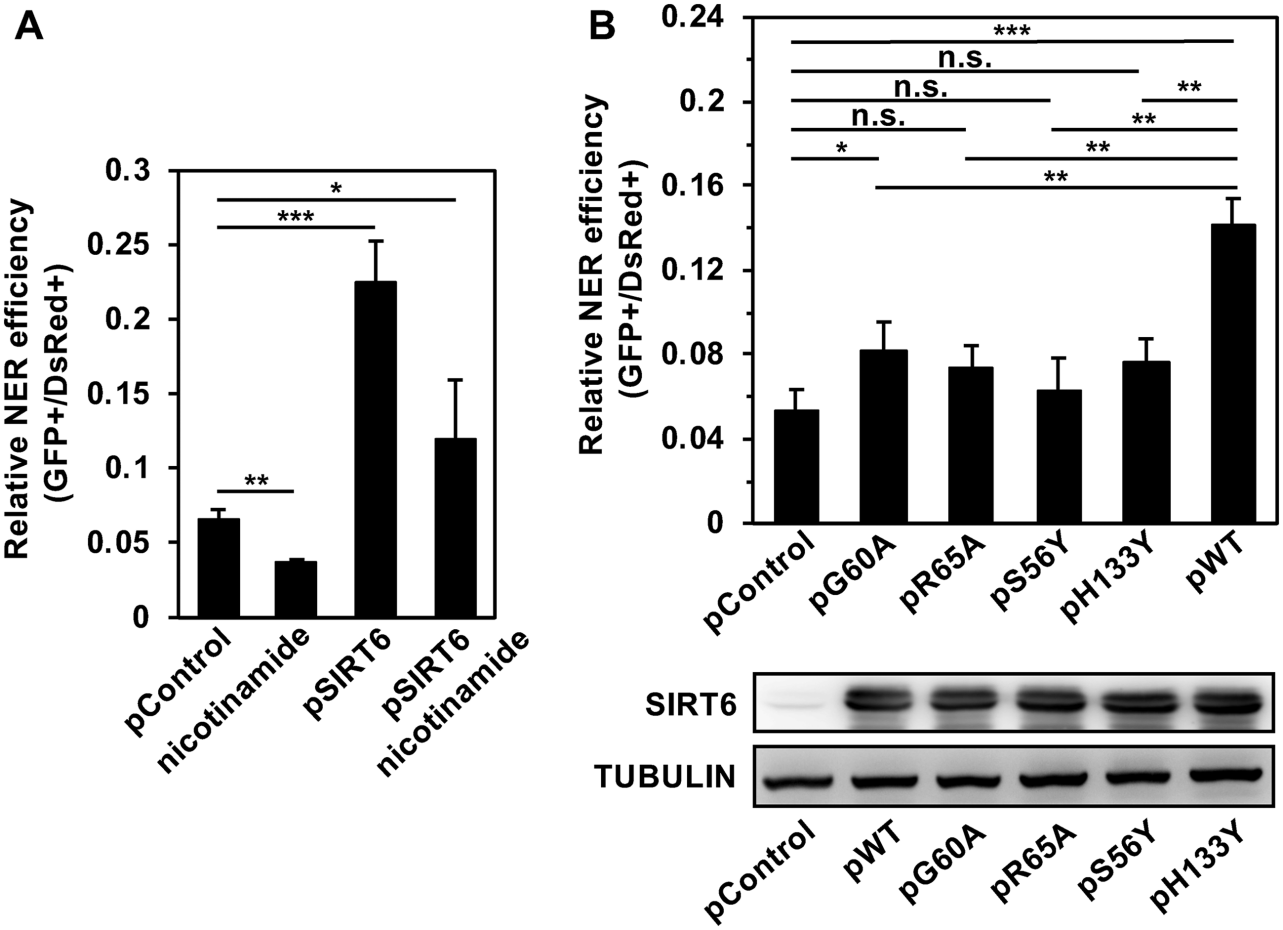
Both enzymatic activities of SIRT6 are required in the repair of UV-induced DNA damage. (**A**) The enhancement of NER efficiency by SIRT6 is compromised by nicotinamide pretreatment. HCA2-hTERT cells were pretreated with nicotinamide at a concentration of 5 mM for 24 h before being transfected with 0.06 μg UVC-treated pmax-GFP and 15 ng pDsRed2-N1. After transfection, the cells were grown in complete medium supplemented with 5 mM nicotinamide for 72 h before being harvested for FACS analysis. Error bars represent the s.d. ***P < 0.001, **P < 0.01, *P < 0.05. (**B**) Inactivating the deacetylation and/or ribosylation activities of SIRT6 with mutations impairs the ability of SIRT6 to promote NER. A control vector or vectors expressing SIRT6 WT or SIRT6 mutants were transfected into HCA2-hTERT together with UVC-treated pmax-GFP and pDsRed2-N1. On day 3 post transfection, the cells were harvested for FACS analysis. The extracted lysates of the cells overexpressing SIRT6 WT or SIRT6 mutants were subjected to western blot analysis. Error bars represent the s.d. ***P < 0.001, **P < 0.01, *P < 0.05, n.s., not significant.

SIRT6 has been demonstrated to regulate DSB repair by mono-ADP-ribosylating PARP1 and promoting the recruitment of SNF2H to DSB sites independent of its enzymatic activities (13,14). We set out to understand whether the regulation of NER by SIRT6 is dependent on its catalytic activities. We pretreated cells with nicotinamide, which is the product of SIRT6-catalyzed reactions and is able to inhibit SIRT6 enzymatic activity by competing with NAD+ to bind to SIRTuin proteins (21), followed by analysis of NER efficiency. We found that the stimulatory effect of SIRT6 overexpression on NER was abolished in the presence of nicotinamide (Figure 3A), suggesting that the regulation of NER by SIRT6 is dependent on its enzymatic activities. In our previous report, we created several SIRT6 mutants to distinguish its deacetylase and mono-ADP-ribosyl transferase activities (13). Among the mutants, SIRT6 G60A retains only deacetylase activity, SIRT6 R65A has only mono-ADP-ribosyl transferase activity, while SIRT6 S56Y and SIRT6 H133Y are enzymatically inactive. By examining the effect of overexpression of each of these mutants on NER efficiency, we found that none of the SIRT6 mutants had the same stimulatory effect as wild-type (WT) SIRT6 (SIRT6 WT) (Figure 3B), suggesting that both enzymatic activities are required for promoting the repair of UV-induced DNA damage.

### SIRT6 promotes GG-NER by targeting DDB2

**Figure 4.**
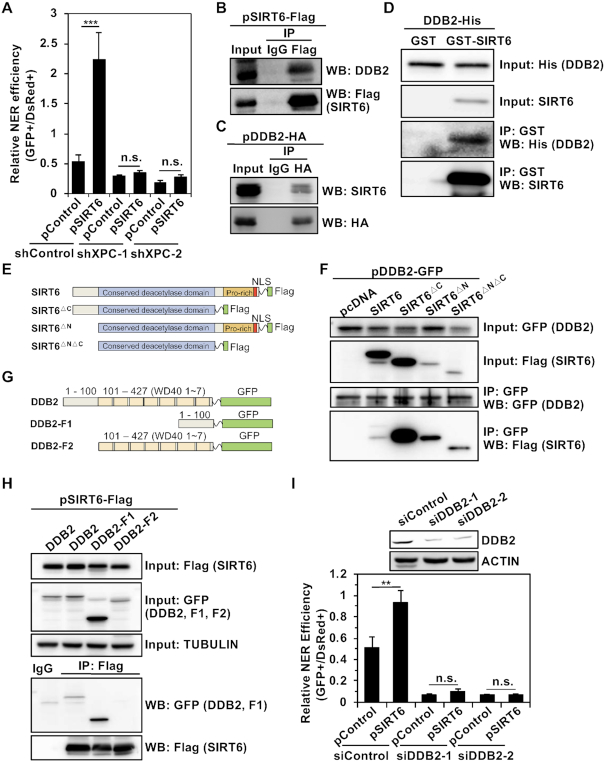
In response to UV irradiation, SIRT6 interacts with DDB2. (**A**) SIRT6 fails to stimulate NER in XPC-depleted HCA2-hTERT cells. A control vector or vector encoding SIRT6 was cotransfected with UVC-treated pmax-GFP and pDsRed2-N1 into control and XPC-depleted HCA2-hTERT cells. At 72 h post transfection, the cells were harvested for FACS analysis. Error bars represent the s.d. ***P < 0.001, n.s., not significant. (B, C) SIRT6 interacts with DDB2 *in vivo*. HEK293 cells were transfected with Flag-tagged SIRT6. At 24 h post transfection, the cells were harvested for immunoprecipitation with an antibody against the Flag tag, followed by western blot analysis with the indicated antibodies (**B**). HEK293 cells were transfected with HA-tagged DDB2. At 24 h post transfection, the cells were harvested for immunoprecipitation with an antibody against the HA tag, followed by western blot analysis (**C**). (**D**) SIRT6 interacts with DDB2 *in vitro*. Five micrograms of recombinant His-DDB2 and GST or GST-SIRT6 together with 30 μl GST-agarose resin were incubated with GST or GST-SIRT6 in IP buffer for 6 h at 4°C. Western blot analysis was performed with the indicated antibodies. (**E**) A schematic representation of the SIRT6 fragments used in this study is shown. (**F**) A control vector or vectors encoding full-length SIRT6-Flag, SIRT6 ΔC-Flag (1-271 aa), SIRT6 ΔN-Flag (49–355 aa) or the deacetylase core fragment SIRT6 ΔNΔC-Flag (49–271 aa) were cotransfected with DDB2-GFP into HEK 293 cells. At 24 h post transfection, the cells were harvested for immunoprecipitation with GFP-Trap (Chromotek), followed by western blot analysis. (**G**) A schematic representation of the DDB2 fragments used in this study is shown. (**H**) Vectors encoding full-length DDB2-GFP, DDB2 fragment 1-GFP (1–100 aa), or DDB2 fragment 2-GFP (101–427 aa) were cotransfected with a SIRT6-Flag-expressing vector into HEK 293 cells. At 24 h post transfection, the cells were harvested for immunoprecipitation with an antibody against Flag, followed by western blot analysis. (**I**) SIRT6 fails to stimulate NER in DDB2-depleted HCA2-hTERT cells. HCA2-hTERT cells were transfected with control siRNA or DDB2-specific siRNA twice over a 48-h interval. Afterwards, the HCA2-hTERT cells were transfected with a control vector or a vector expressing SIRT6 and UVC-treated pmax-GFP together with pDsRed2-N1. On day 3 post transfection, the cells were harvested for FACS analysis. Depletion of DDB2 from fibroblasts was confirmed by western blot analysis. Error bars represent the s.d. **P < 0.01, n.s., not significant.

The NER pathway can be further categorized into two subpathways, GG-NER and TC-NER, which differ in the initiation steps. To examine which subpathway is regulated by SIRT6, we examined the stimulatory effect of SIRT6 on cells with XPC, a GG-NER factor, depleted. We found that depleting XPC led to the abrogation of the stimulatory effect (Figure 4A), suggesting that SIRT6 is involved in the process of GG-NER.

We then performed co-IP experiments to examine whether SIRT6 interacts with any of the GG-NER initiation factors, including XPC, XPA, DDB1 and DDB2. We found that DDB2 but no other factor tested interacted with SIRT6 *in vivo* (Figure 4B, C, Supplementary Figure S5). *In vitro* co-IP experiments with recombinant SIRT6 and DDB2 confirmed that these two proteins interacted with each other (Figure 4D). In addition, we ruled out the possibility that the interaction between SIRT6 and DDB2 was mediated by DNA, as we found that pretreatment with ethidium bromide did not abolish the interaction between SIRT6 and DDB2 (Supplementary Figure S6). Moreover, we confirmed that SIRT6 interacted with DDB2 in the melanoma cell line A875 (Supplementary Figure S7A, B).

Further co-IP experiments demonstrated that removing either the C- or N-terminal domains of SIRT6 or both did not abrogate the interaction between SIRT6 and DDB2 (Figure 4E, F), indicating that the central catalytic domain of SIRT6 interacts with DDB2. We separated full-length DDB2 into two fragments, DDB2-F1 (1–100 aa) and DDB2-F2 (101–427 aa), with DDB2-F2 containing seven WD40 domains (22), and examined which fragment of DDB2 interacts with SIRT6. The results indicated that DDB2-F1 (1–100 aa) interacted with SIRT6 (Figure 4G, H).

We then tested whether SIRT6 promotes GG-NER by targeting DDB2. Using the plasmid reactivation assay for measuring NER efficiency, we found that depleting DDB2 completely abolished the stimulatory effect of SIRT6 overexpression on NER (Figure 4I).

SIRT6 promotes both DSB repair and BER in a PARP1-dependent manner (13,17). Recently, it has been proposed that PARP1 is involved in NER (23). We therefore examined whether SIRT6 regulates NER by activating PARP1. We found that inhibiting PARP1 with the enzymatic inhibitor PJ34 did not abolish the stimulatory effect of SIRT6 on NER (Supplementary Figure S8), suggesting that SIRT6 regulates NER in a PARP1-independent manner. SIRT6 has also been reported to interact with PKM2 (24), which may participate in NER by interacting with DDB2 upon UV irradiation (25). To rule out the possibility that SIRT6 regulates NER through PKM2, we knocked down PKM2 expression and then examined the stimulatory effect of SIRT6 on NER. We found that depleting PKM2 did not abrogate the increase in NER efficiency observed with SIRT6 overexpression (Supplementary Figure S9A, B). In addition, we did not observe any change in the interaction between DDB1 and DDB2 in SIRT6-depleted cells (Supplementary Figure S9C).

Thus, we conclude that SIRT6 promotes GG-NER to repair UV-induced DNA damage by targeting DDB2.

### SIRT6 deacetylates DDB2 in response to UV irradiation

**Figure 5.**
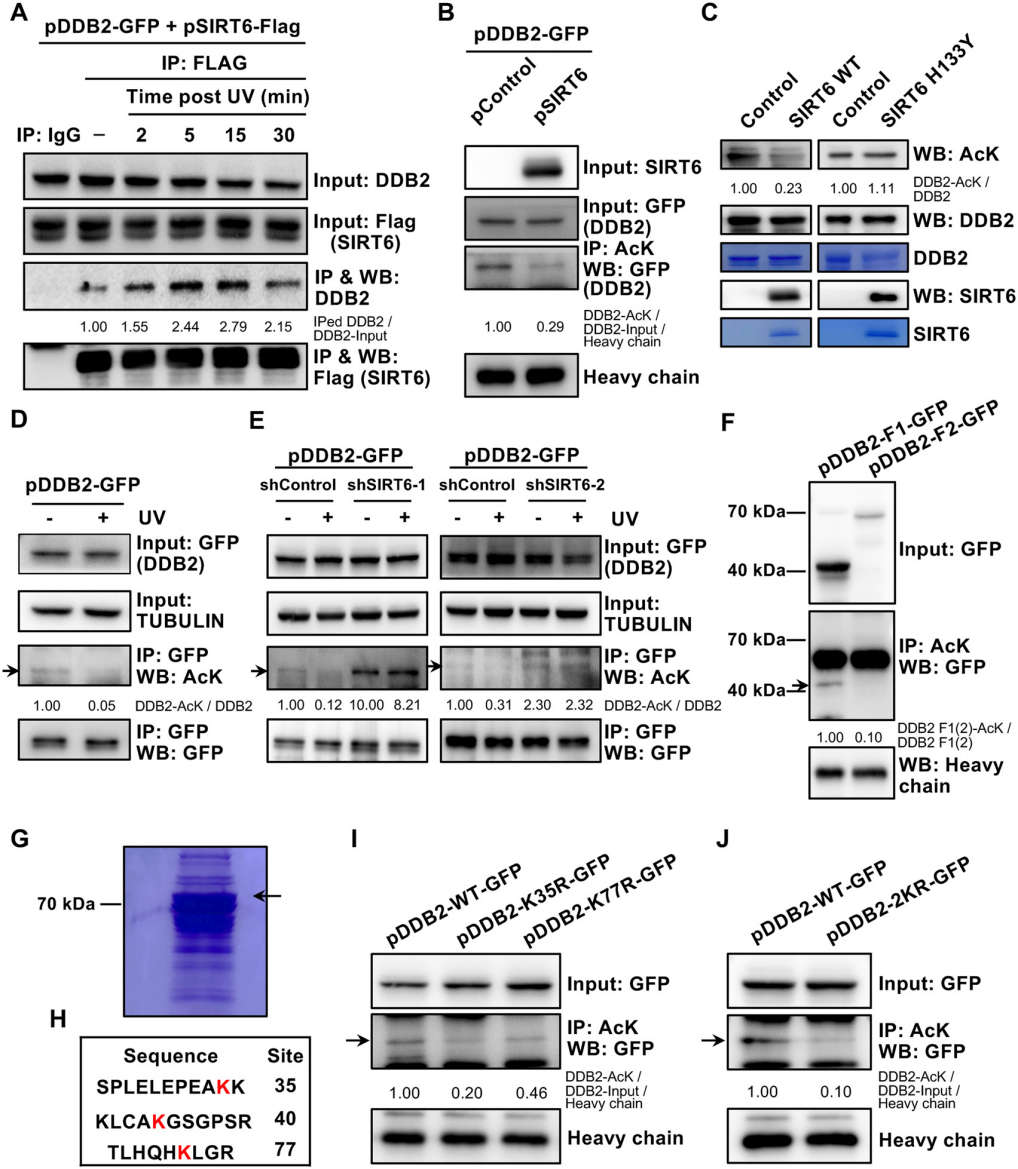
SIRT6 deacetylates DDB2 in response to UV irradiation. (**A**) The interaction between SIRT6 and DDB2 is enhanced in response to UVC irradiation. HEK293 cells with stable Flag-tagged SIRT6 integration were transfected with DDB2-GFP and treated with or without UVC (20 J/m^2^). Then, cells were harvested at the indicated time points. Cell lysates were immunoprecipitated with an anti-Flag antibody, followed by western blot analysis with the indicated antibodies. IPed, immunoprecipitated. (**B**) DDB2 is a target of the deacetylase SIRT6. HEK293 cells were cotransfected with plasmids encoding DDB2-GFP and SIRT6-Flag. At 16 h post transfection, the cells were harvested for immunoprecipitation with an antibody against acetylated lysines (AcK), followed by western blot analysis with the indicated antibodies. (**C**) DDB2 is deacetylated by SIRT6 *in vitro*. A recombinant DDB2-GFP protein (5 μg) and a SIRT6 WT or H133Y mutant protein (5 μg) purified from HEK293 cells were incubated to allow the deacetylation reaction to occur in HDAC buffer (50 mM Tris-HCl pH 9.0, 4 mM MgCl_2_, 50 mM NaCl, 0.2 mM DTT and 1 mM NAD+) at 30°C for 2.5 h (41). Then, the reactions were analyzed by western blot analysis using antibodies against AcK, DDB2 and SIRT6. The recombinant DDB2-GFP, SIRT6 WT or SIRT6 H133Y mutant proteins were also resolved on SDS-PAGE gels, followed by Coomassie Blue staining. The gel was stained with Coomassie reagent (ratio, methanol:acetic acid:Coomassie:H_2_O = 45:10:0.25:45) for 2.5 h, followed by washing with a destaining solution (ratio, methanol:acetic acid:H_2_O = 25:8:67). (**D**) The change in the acetylation level of exogenous DDB2 in response to UV irradiation was evaluated. HCA2-hTERT cells were transfected with a vector encoding DDB2-GFP and irradiated with or without UVC (20 J/m^2^). Then, the cells were harvested at 5 min post UV irradiation. Cell lysates were immunoprecipitated with GFP-Trap, followed by western blot analysis with the indicated antibodies. The arrow indicates the acetylated DDB2-GFP. (**E**) The changes in the acetylation levels of exogenous DDB2 in control and SIRT6-depleted cells in response to UV irradiation were evaluated. The control and SIRT6-depleted HCA2-hTERT cells were transfected with a vector encoding DDB2-GFP and irradiated with or without UVC (20 J/m^2^). Then, the cells were harvested at 5 mins post UV irradiation. Cell lysates were immunoprecipitated with GFP-Trap, followed by western blot analysis with the indicated antibodies. The arrow indicates the acetylated DDB2-GFP. (**F**) The acetylation levels of DDB2 fragments were assessed. HEK293 cells were transfected with vectors encoding the GFP-tagged DDB2 fragments, DDB2 fragment 1-GFP (1–100 aa) or DDB2 fragment 2-GFP (101–427 aa). At 16 h post transfection, the cells were harvested for immunoprecipitation with an antibody against acetylated lysine, followed by western blot analysis. The arrow indicates the acetylated DDB2-F1-GFP. (**G**) The Coomassie blue-stained SDS-PAGE gel image shows that DDB2 immunoprecipitated from HEK293 cells with an anti-GFP antibody. HEK293 cells were transfected with a plasmid encoding DDB2-GFP. At 16 h post transfection, the cells were harvested for immunoprecipitation with an antibody against GFP, followed by SDS-PAGE. (**H**) A schematic representation of lysine residues in DDB2 fragment 1 (1–100 aa) is shown. (**I**) K35 and K77 are the two lysine residues that are acetylated in DDB2. DDB2-GFP WT and the indicated DDB2 mutants were transfected into HEK293 cells. GFP-tagged DDB2 was pulled down from cell lysates using an anti-acetylated lysine antibody and immunoblotted with the indicated antibodies. The arrow indicates the acetylated DDB2-GFP WT or mutants. (**J**) The acetylation levels of DDB2-GFP WT and the 2KR mutant were measured. DDB2-GFP WT and DDB2-GFP 2KR were transfected into HEK293 cells. GFP-tagged DDB2 was pulled down from cell lysates using an anti-acetylated lysine antibody and immunoblotted with the indicated antibodies. The arrow indicates the acetylated DDB2-GFP WT or mutant.

To test whether SIRT6 promotes NER by deacetylating or mono-ADP-ribosylating DDB2, we first examined changes in the interaction between SIRT6 and DDB2 upon UV irradiation. We found that the interaction between SIRT6 and DDB2 was enhanced after UV irradiation (Figure 5A). In contrast, the interaction between the enzymatically dead mutant SIRT6 H133Y and DDB2 did not increase in response to UV irradiation (Supplementary Figure S10). These data indicate that modification of DDB2 by SIRT6 is critical in NER.

To test whether DDB2 is deacetylated by SIRT6, we first examined whether DDB2 is acetylated in cells. We observed that the DDB2 acetylation level was enhanced in cells overexpressing CBP and GCN5, two acetyltransferases (Supplementary Figure S11), suggesting that DDB2 may be a target of these two acetyltransferases. We then performed experiments to examine whether SIRT6 deacetylates DDB2. We found that overexpressing SIRT6 but not the enzymatically dead mutant SIRT6 H133Y promoted the deacetylation of DDB2 (Figure 5B, Supplementary Figure S12), which reduced the acetylation level of DDB2. A further *in vitro* deacetylation assay confirmed that SIRT6 WT but not the SIRT6 H133Y mutant deacetylated DDB2 (Figure 5C). Moreover, we found that UV irradiation led to a decrease in the acetylation level of exogenous DDB2 or endogenous DDB2 in control cells, while depleting SIRT6 abolished the UV-induced declines in both exogenous and endogenous DDB2 acetylation levels (Figure 5D, E, Supplementary Figure S13A, B). In addition, in SIRT6-depleted cells, the acetylation level of DDB2 was increased (Figure 5E, Supplementary Figure S13C). Since both the deacetylase activity and mono-ADP-ribosyl transferase activity of SIRT6 are required for its function in NER, we also tested whether the SIRT6 R65A mutant, which has mono-ADP-ribosyl transferase activity but not deacetylase activity, interacts with DDB2 upon UV irradiation. We did not observe any enhancement of the interaction between the SIRT6 R65A mutant and DDB2 (Supplementary Figure S14), indicating that the regulation of NER by SIRT6 probably does not occur through mono-ADP-ribosylation of DDB2. Taken together, our results demonstrate that SIRT6 regulates NER by deacetylating DDB2.

To identify which lysine residues on DDB2 are deacetylated by SIRT6, we first tested which of the two fragments DDB2-F1 (1–100 aa) and DDB2-F2 (101–427 aa) was acetylated. Co-IP experiments demonstrated that DDB2-F1 (1–100 aa) was acetylated (Figure 5F). We then immunoprecipitated DDB2 and analyzed potential acetylated lysine residues through mass spectrometry. We identified three lysine sites in DDB2-F1 (1–100 aa) (K35, K40 and K77) that were acetylated (Figure 5G, H, Supplementary Figure S15A). We then created the DDB2 mutants K35R, K40R and K77R and examined the acetylation level of each of the three mutants. We found that mutating K35 or K77 but not K40 diminished the acetylation level of DDB2 (Figure 5I, Supplementary Figure S15B). We also observed a drastic reduction in the acetylation level of the DDB2 2KR mutant, which contained both the K35R mutation and the K77R mutation (Figure 5J).

To further confirm whether the K35 and K77 are the relevant lysine residues, we analyzed the change in the acetylation level of DDB2 in control and SIRT6-depleted cells. We found that the acetylation level of the DDB2 2KR mutant did not change in SIRT6-depleted cells (Supplementary Figure S16), further indicating that the acetylation occurs on the two lysine residues.

### Depleting SIRT6 leads to the retention of DDB2 at chromatin in response to UV-induced DNA damage

To understand the consequences of the SIRT6-mediated deacetylation of DDB2, we first examined the expression levels of DDB2 in SIRT6-overexpressing or SIRT6-depleted cells. We found that neither overexpression nor depletion of SIRT6 affected the overall DDB2 protein level under normal conditions (Supplementary Figure S17).

**Figure 6.**
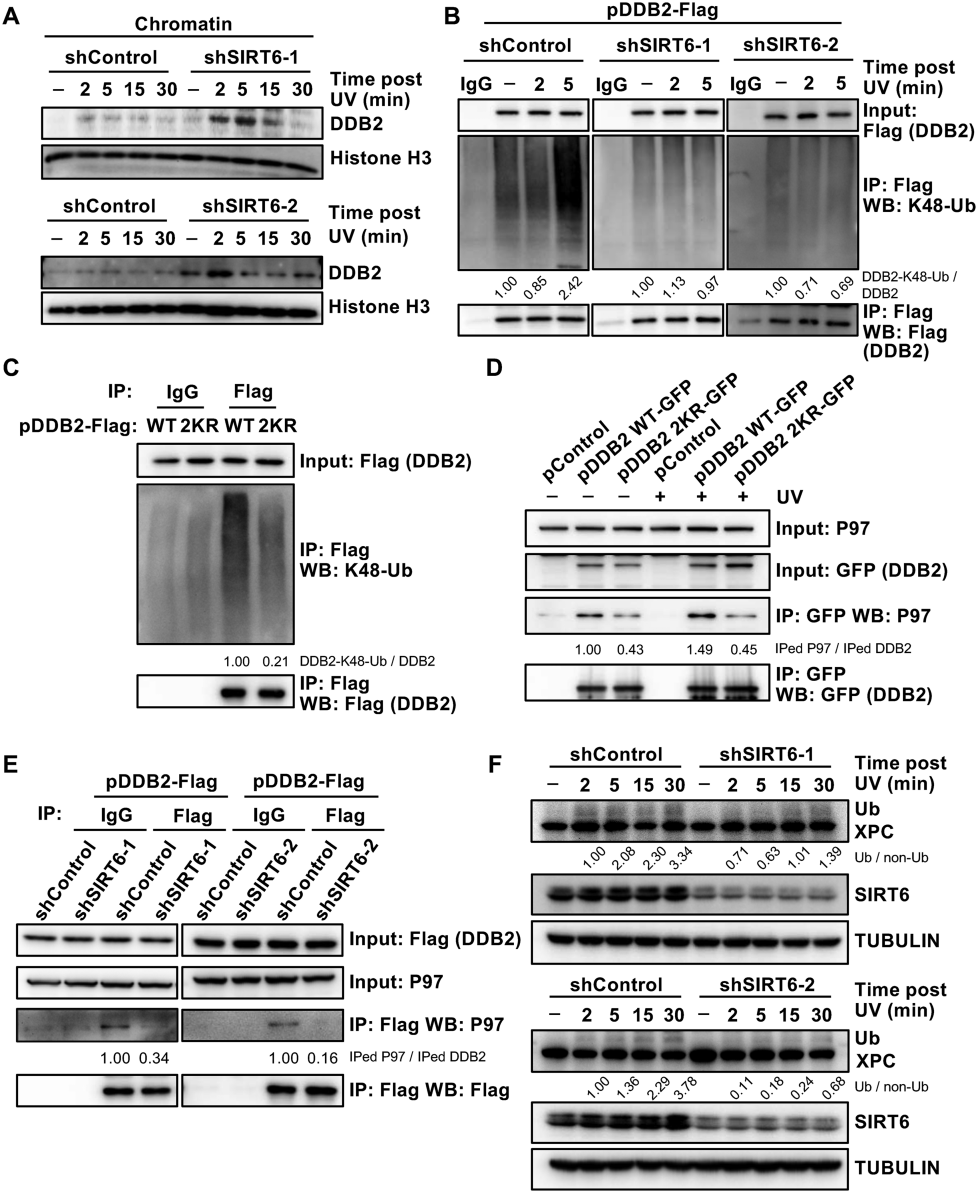
Loss of SIRT6 leads to the retention of DDB2 on chromatin in response to UV irradiation. (**A**) Changes in DDB2 bound to chromatin in the absence of SIRT6 in response to UV irradiation were evaluated. Control and SIRT6-depleted HCA2-hTERT cells were irradiated with UVC (20 J/m^2^), harvested at the indicated time points and subjected to cellular protein fractionation, followed by western blot analysis of DDB2 in the chromatin fractions. (**B**) Depleting SIRT6 abolished the increased ubiquitination of DDB2 upon UV irradiation. Control and SIRT6-depleted HEK293 cells were transfected with a plasmid encoding DDB2-Flag and treated with MG132 at 10 μM. At 16 h post transfection, the cells were harvested for immunoprecipitation with an antibody against Flag, followed by western blot analysis with an antibody recognizing ubiquitin. (**C**) The ubiquitination level of the DDB2 2KR mutant was lower than that of DDB2 WT. HEK293 cells were transfected with a DDB2-Flag or DDB2-2KR-Flag plasmid. After 16 h, the cells were harvested for immunoprecipitation with an antibody against Flag, followed by western blot analysis with an antibody recognizing ubiquitin. (**D**) DDB2 2KR partially abolished the interaction between DDB2 and p97. HEK293 cells were transfected with a pControl, DDB2-GFP or DDB2-2KR-GFP plasmid. After 16 h, the cells were irradiated with UVC or left untreated, and at 5 min post UV irradiation, the cells were harvested for immunoprecipitation with an antibody against GFP and immunoblotted with the indicated antibodies. IPed, immunoprecipitated. (**E**) SIRT6 greatly diminished the interaction between DDB2 and p97. Control and SIRT6-depleted HEK293 cells were transfected with a plasmid encoding DDB2-Flag. After 16 h, the cells were harvested for immunoprecipitation with an antibody against Flag and immunoblotted with the indicated antibodies. IPed, immunoprecipitated. (**F**) Immunoblot analysis of changes in XPC hyperubiquitination in the absence or presence of SIRT6 was performed. Control and SIRT6-depleted HCA2-hTERT cells were UVC irradiated (20 J/m^2^) and harvested at the indicated time points, followed by western blot analysis.

Upon UV-induced DNA damage, the rapid recruitment of DDB2 to DNA damage sites facilitates the recruitment of XPC to these lesions, thereby initiating the GG-NER signaling cascade (26). Subsequently, DDB2 is rapidly ubiquitinated and removed from chromatin to accelerate the repair process (27). We therefore set out to examine whether changes in the levels of chromatin-associated DDB2 occur upon UV-induced DNA damage, and we observed that SIRT6 depletion caused the retention of DDB2 on chromatin (Figure 6A).

Given that SIRT6 mediates the UV-induced deacetylation of DDB2 and that acetylation is the major mechanism preventing ubiquitination (28), we hypothesized that in response to UV irradiation, SIRT6 deacetylates DDB2 to promote DDB2 ubiquitination and segregation from chromatin. To test this hypothesis, we analyzed the ubiquitination level of DDB2 in control and SIRT6-depleted cells upon UV irradiation. In agreement with a previous report (27), the ubiquitination level of DDB2 was increased at 5 min post UV treatment in control cells (Figure 6B). In contrast, the increase in the ubiquitination level of DDB2 was not seen in SIRT6-depleted cells (Figure 6B). Moreover, we also found that the ubiquitination level of the DDB2 2KR mutant was lower than that of DDB2 WT (Figure 6C). In addition, in cells overexpressing the enzymatically dead SIRT6 H133Y mutant, the ubiquitination level of DDB2 was lower than that in cells overexpressing SIRT6 WT (Supplementary Figure S18). These results indicate that the increase in the ubiquitination level of DDB2 is at least partially dependent on SIRT6 deacetylase activity.

Ubiquitinated DDB2 has a high affinity for p97, the segregase that removes it from damaged DNA sites (27). Therefore, we examined whether SIRT6 affects the interaction between DDB2 and p97 upon UV irradiation. We observed that two mutations in DDB2 (K35R and K77R) partially abolished the interaction between DDB2 and p97 and suppressed the UV-induced enhancement of the interaction between these molecules (Figure 6D). Moreover, we found that SIRT6 depletion greatly diminished the interaction between DDB2 and p97 (Figure 6E).

These data indicate that in response to UV irradiation, SIRT6 deacetylates DDB2 at two lysine residues, K35 and K77, promoting the ubiquitination of DDB2. Subsequently, the affinity between DDB2 and p97 is enhanced, and ubiquitinated DDB2 is segregated from damaged DNA to facilitate the subsequent NER signaling cascade. Indeed, as a consequence, we observed that less XPC was ubiquitinated in SIRT6-depleted cells than in control cells (Figure 6F) (29), strongly indicating that the absence of SIRT6 impairs the signaling cascade involved in GG-NER initiation.

### SIRT6 mutations in melanoma patients contribute to a loss of NER efficiency

**Figure 7.**
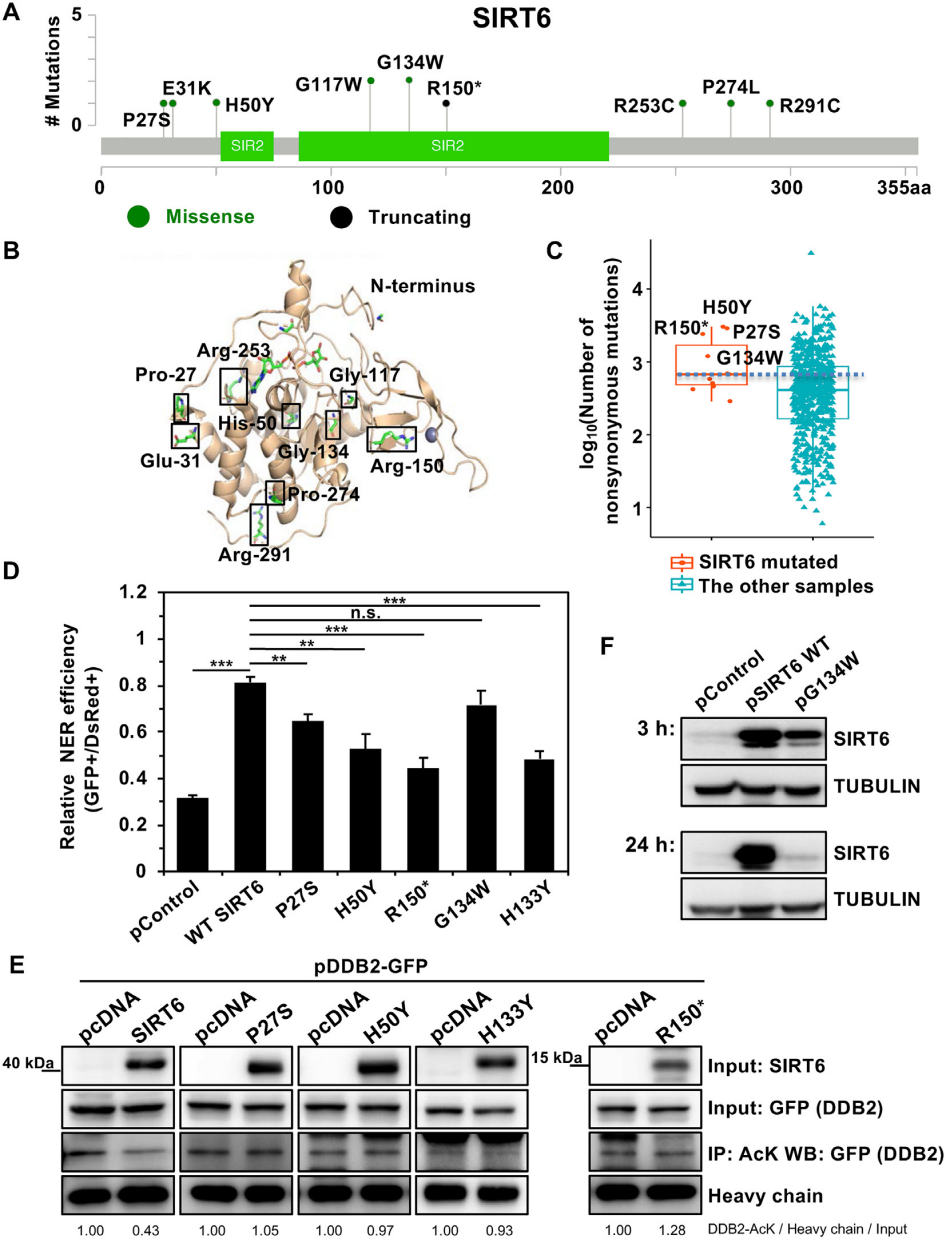
Several mutations in SIRT6 in melanoma impair the repair of UV-induced DNA damage and result in a high incidence of mutation rates across the genome. (**A**) Lolliplot of the SIRT6 protein with the alterations present in melanoma samples indicated. (**B**) Locations of the alterations mapped to the SIRT6 crystal structure (PDB: 3ZG6). (**C**) Number of nonsynonymous mutations in melanoma samples with SIRT6 mutations. (**D**) Several SIRT6 mutants lost the ability to enhance NER efficiency. Error bars represent the s.d. ***P < 0.001, **P < 0.01, n.s., not significant. (**E**) The SIRT6 P27S and H50Y mutants partially lost their deacetylase activity. HEK293 cells were cotransfected with plasmids encoding DDB2-GFP and SIRT6 WT or mutants. At 16 h post transfection, the cells were harvested for immunoprecipitation with an antibody against acetylated lysine, followed by western blot analysis with the indicated antibodies. (**F**) SIRT6 G134W has a high turnover rate. HCA2-hTERT cells were harvested for protein extraction at 3 and 24 h post transfection with a control vector, SIRT6 WT or G134W mutant, followed by western blot analysis of SIRT6 expression.

To determine if naturally occurring mutations in SIRT6 in melanoma patients can inactivate the NER pathway, thereby promoting tumorigenesis, we identified nine melanoma patient-derived SIRT6 somatic mutations from COSMIC, variations called from whole-exome sequencing of TCGA samples and variations called from whole-genome sequencing of ICGC samples (Figure 7A, B). We then counted the nonsynonymous mutation numbers of the TCGA and ICGC melanoma patients and found that among them, four mutations in SIRT6, P27S, H50Y, R150* and G134W, correlated with high mutation rates across the genome (Figure 7C).

We created vectors expressing the four SIRT6 mutants by performing mutagenesis experiments. By overexpressing these mutants, we found that three mutants (P27S, H50Y, and R150*) at least partially abolished the stimulatory effect on NER in both HCA2-hTERT cells and A875 cells (Figure 7D, Supplementary Figure S19A). R150* is a nonsense mutant; therefore, it was not surprising to observe the loss of stimulation. We then set out to understand how SIRT6 P27S and SIRT6 H50Y abrogate the stimulatory effect on NER. We found that overexpressed SIRT6 P27S and SIRT6 H50Y failed to deacetylate DDB2 and H3K56Ac (Figure 7E, Supplementary Figure S19B), suggesting that the P27S or H50Y mutation in SIRT6 impairs its function in NER by eliminating SIRT6 deacetylase activity. Moreover, we demonstrated that the SIRT6 mutants failed to promote the removal of DDB2 from chromatin upon UV stress (Supplementary Figure S19C).

However, puzzlingly, the G134W mutation in SIRT6 was also associated with high mutation rates in the melanoma patients, but it did not abolish the stimulatory effect of SIRT6 on NER in HCA2-hTERT cells. We hypothesized that although this mutant retains its normal function in NER, the turnover rate of SIRT6 G134W might be high. Indeed, we found that SIRT6 G134W expression was high at 3 h post transfection, but the expression had declined dramatically by 24 h post transfection (Figure 7F). These data indicate that rapid but transient overexpression is sufficient to boost NER efficiency, as assayed by the plasmid reactivation method in HCA2-hTERT cells, but endogenous mutation of SIRT6 leads to low levels of SIRT6 and eventually destabilizes the genome in melanocytes.

## DISCUSSION

SIRT6 is a critical factor regulating longevity, as SIRT6-deficient mice exhibit a premature aging phenotype, while overexpression of SIRT6 significantly extends the lifespan of male mice (16,30). The incidence of nearly all types of cancers increases with age in humans (31), and SIRT6 has been characterized as a tumor suppressor in several organs (32). Although its tumor suppressive functions have been mainly focused on transcriptional regulation, stabilizing genomes through activating different DNA repair pathways probably contributes to the suppression of tumorigenesis, thereby extending the lifespan. The role of SIRT6 in regulating DSB repair has been extensively investigated, but here, for the first time, we demonstrate that SIRT6 is also a positive regulator of NER. After demonstrating that several clinically relevant mutations in SIRT6 cause NER deficiency and are present in tumors with high mutation rates across the genome, we propose that SIRT6 is a tumor suppressor during melanomagenesis.

However, interestingly, a previous report indicated that SIRT6 acted as an oncogene by promoting the expression of the prosurvival factor COX2 in human epidermal keratinocytes (33). As a consequence, SIRT6 expression is upregulated in human skin squamous cell carcinoma, the second most common type of skin cancer. These seemingly opposing functions of SIRT6 in two types of skin cancers may be explained by differences in cell types or context, as previously reviewed (32). Another possibility is that SIRT6 may act as a double-edged sword during skin tumorigenesis. In response to DNA damage induced by UV irradiation, SIRT6 may exert a tumor-suppressive function by stabilizing the genome through upregulating NER. Deregulation of SIRT6 may result in high genomic mutation rates, thereby increasing the risk of mutating tightly controlled pro-oncogenes and tumor suppressor genes, eventually giving rise to tumorigenesis. Once a tumor forms, tumor cells can take advantage of the prosurvival function of SIRT6 to suppress AMPK phosphorylation and promote COX2 expression. Nevertheless, whether SIRT6 promotes DNA repair by NER in keratinocytes and whether SIRT6 promotes COX2 expression in human melanocytes need to be further investigated.

As a deacetylase, SIRT6 catalyzes deacetylation reactions on both histones, including H3K9Ac, H3K56Ac and H3K18Ac (34–36), and non-histone substrates, such as PKM2, GCN5 and p53 (24,37,38). Here, we identified another non-histone protein, DDB2, as a deacetylation substrate of SIRT6. SIRT6 mediated the deacetylation of DDB2 on the two lysine residues K35 and K77 and subsequent segregation of DDB2 from DNA lesions in response to UV irradiation, thereby facilitating the process of NER.

In addition, our data suggest that the mono-ADP-ribosyl transferase activity of SIRT6 is also required for repairing UV-induced DNA damage. Previous research has successfully identified several targets of SIRT6, such as mono-ADP-ribosyl transferase, PARP1, KAP1 and BAF170 (13,39,40), among which PARP1 is a critical factor involved in nearly all types of DNA repair. However, we ruled out the possibility that SIRT6 targets PARP1 to regulate NER. Which protein is the target of SIRT6 mono-ADP-ribosyl transferase activity and how SIRT6 regulates NER to preserve genome integrity by modifying this target remain to be further determined.

Our data mining and experiments indicate that two different mutations in SIRT6 can cause the loss of enzymatic activity, a frame shift or high turnover rates and thereby negatively impact NER and genomic stability. Other mutations in SIRT6 may be further categorized into two groups. One group of mutations may be neutral, and melanomagenesis in these patients could be driven by other factors. The other group of mutations may lead to deregulation of other tumor-suppressive functions of SIRT6. For instance, these mutations may alter the transcription of essential genes involved in the ‘Warburg effect’ or downregulate DSB repair and destabilize telomere structures. However, further investigation is still warranted to understand the potential roles of these mutations in melanoma.

In summary, our study demonstrates that SIRT6 is a critical tumor suppressor in melanomagenesis that functions by promoting the repair of UV-induced DNA damage through GG-NER. Mechanistically, upon UV irradiation, SIRT6 binds to DNA damage sites and deacetylates two lysine residues, K35 and K77, on DDB2, promoting DDB2 ubiquitination and segregation from chromatin, which ultimately facilitates the NER signal transduction cascade (Supplementary Figure S20). Our work strongly suggests that activating SIRT6 may help stabilize the genome to prevent UV-induced melanomagenesis

## DATA AVAILABILITY

The mass spectrometry proteomics data have been deposited to the ProteomeXchange Consortium (http://proteomecentral.proteomexchange.org) via the iProX partner repository with the dataset identifier PXD020721.

## Supplementary Material

gkaa661_Supplemental_File
