# Supplementary material for: The deacetylase SIRT6 promotes the repair of UV-induced DNA damage by targeting DDB2
Source: Nucleic Acids Res. 2020 Aug 13;48(16):9181–94. doi: 10.1093/nar/gkaa661 (PMC7498349; doi:10.1093/nar/gkaa661)
Supplement: gkaa661_Supplemental_File [file gkaa661_supplemental_file.pdf]

Supplementary Materials and Methods

LC-MS/MS analysis and data processing

Experiments were performed by PTM Biolab (Hangzhou, China). For in-gel tryptic digestion, gel pieces were destained in 50% acetonitrile containing 50 mM NH_4HCO_3, followed by dehydration with 100% acetonitrile for 5 min. After the acetonitrile was removed, the samples were incubated in a 10 mM dithiothreitol solution at 37°C for 60 min, followed by another round of dehydration. Then, the samples were incubated in a 55 mM iodoacetamide solution at room temperature for 45 min in the dark. After washing with a 50 mM NH_4HCO_3 solution, the samples were incubated with 100% acetonitrile for dehydration. The dehydrated samples were then resuspended in 50 mM NH_4HCO_3 containing 10 ng/μl trypsin and further incubated on ice for 1 h. The cleaned samples were then trypsinized overnight at 37°C, followed by peptide extraction with 50% acetonitrile/5% formic acid and 100% acetonitrile. The extracted peptides were then subjected to LC-MS/MS analysis.

For LC-MS/MS analysis, the peptides were dissolved in solvent A (0.1% formic acid) and separated by an EASY-nLC 1000 UPLC system by direct loading onto a reversed-phase analytical column (Acclaim PepMap 100, Thermo Fisher Scientific, Waltham, MA, USA). The gradient consisted of different concentrations of solvent B (0.1% formic acid in 90% acetonitrile) with a constant flow rate of 500 nl/min: 6%-35% for 0-22 min, 35%-80% for 22-26 min and 80% solvent B for 26-30 min.

After separation by ultra-performance liquid chromatography, the peptides were sent to a nanospray ionization (NSI) source, followed by tandem mass spectrometry (MS/MS) on a Q ExactiveTM Plus (Thermo Fisher Scientific, Waltham, MA, USA). The electrospray voltage was set to 2.1 kV. The full scan was set to a range of 350 to 1,800. The intact peptides and fragments were detected in the Orbitrap at resolutions of 70,000 and 17,500, respectively. Data were acquired by a data-independent acquisition (DDA) program. The top 20 precursor ions were sequentially sent to a high collision dissociation cell (HCD) and dissociated by 28% collision energy. Automatic gain control (AGC) was specified as 5E4. The signal threshold was set to 5,000 ions/s. The maximum injection time was set to 200 ms, and the dynamic exclusion time was set to 15 s.

The MS/MS data were analyzed by Proteome Discoverer (Ver. 1.3) and were searched against the DDB2 protein sequence. The cleavage enzyme was specified to be trypsin/P. Up to 4 missing cleavage fragments were allowed. Mass errors for precursor ions and fragment ions were set to 10 ppm and 0.02 Da. Carbamidomethyl on Cys was set as a fixed modification. Acetylation modification on the Tyr residues and N terminus of proteins and oxidation on Met were set as variable modifications. The peptide ion score and peptide confidence were specified as high and >20, respectively.

The major peptides are provided in Fig. 5H and Supplementary Figure 15.

Supplementary Figure Legends

Supplementary Figure 1. Related to Figure 1. Validation of the plasmid reactivation assay for measuring NER efficiency. **(A)** FACS analysis of GFP+ cells in samples transfected with pmax-GFP

plasmids irradiated with increasing doses of UVC. **(B-C)** Validation of the plasmid reactivation assay for measuring NER efficiency. Control or XPC-depleted or CSA-depleted fibroblasts were transfected with 0.06 µg UVC-treated pmax-GFP together with 15 ng pDsRed2-N1 as an internal control for normalizing transfection efficiency. The ratio of GFP+ cells to DsRed+ cells was employed as the measure of NER efficiency. Western blot analysis of XPC **(B)** or CSA **(C)** expression levels in control and XPC-knockdown or CSA-knockdown cells was performed. All experiments were repeated at least three times. Error bars represent the s.d. ** $P < 0.01$, **** $P < 0.0001$. **(D)** UVC irradiation did not lead to linearization of the pmax-GFP plasmid. The pmax-GFP plasmid was digested with the AgeI restriction enzyme or irradiated with UVC, followed by electrophoresis on an agarose gel.

Supplementary Figure 2. Related to Figure 1. NER efficiency in SIRT6-knockout MEFs and melanoma cells. **(A)** The efficiency of NER in SIRT6-knockout MEFs. The analysis of NER efficiency was performed as described in Fig. 1B. All experiments were repeated at least three times. Error bars represent the s.d. ** $P < 0.01$. **(B)** Western blot analysis of SIRT6 in control and SIRT6-knockout cells. **(C)** Effect of SIRT6 depletion on NER in the melanoma cell line A875. Control and SIRT6-depleted A875 cells were transfected with 0.06 µg UVC-treated pmax-GFP together with 15 ng pDsRed2-N1 as an internal control for normalizing transfection efficiency. On day 3 post transfection, cells were harvested for FACS analysis. The ratio of GFP+ cells to DsRed+ cells was employed as the measure of NER efficiency. All experiments were repeated at least three times. Error bars represent the s.d. ** $P < 0.01$. **(D)** Western blot analysis of SIRT6 in control and SIRT6-knockdown cells. **(E)** Effect of SIRT6 overexpression on NER. A875 cells were transfected with 0.06 µg UVC-treated pmax-GFP together with 15 ng pDsRed2-N1 and 5 µg control vector or vector encoding SIRT6, followed by FACS analysis on day 3 post transfection. All experiments were repeated at least three times. Error bars represent the s.d. *** $P < 0.001$. **(F)** Western blot analysis of SIRT6 overexpression in the A875 cell line.

Supplementary Figure 3. Related to Figure 1. Representative images of U2OS cells showing the recruitment of GFP–SIRT6 and GFP-KU70 to sites of DNA damage induced by laser microirradiation (with or without 8-mop). **(A)** U2OS cells were transfected with a GFP-KU70 plasmid. At 24 h post transfection, the cells were irradiated with a laser. **(B)** U2OS cells were transfected with 1 µg GFP-KU70 plasmid. At 24 h post transfection, the cells were irradiated with a laser and immunostained with anti-CPD and anti-GFP antibodies. **(C)** U2OS cells were transfected with a GFP-SIRT6 plasmid. At 24 h post transfection, the cells were irradiated with a laser. The analysis of laser microirradiation was performed as described in the Materials and Methods section.

Supplementary Figure 4. Related to Figure 2. Depleting SIRT6 inhibits cell proliferation in response to UVC irradiation in A875 cells. A875 cells were exposed to 20 J/m^2 UVC, followed by cell counting at 72 h, 96 h and 120 h post UV irradiation. *** $P < 0.001$, ** $P < 0.01$.

Supplementary Figure 5. Related to Figure 4. SIRT6 does not interact with other factors that initiate NER. The experimental procedure is described in Fig. 5A. HEK293 cells with stable Flag-tagged SIRT6 integration were treated with UVC (20 J/m^2) or left untreated. Then, the cells were harvested at the indicated time points. Cell lysates were immunoprecipitated with an anti-Flag antibody, followed by Western blot analysis with the indicated antibodies.

Supplementary Figure 6. Related to Figure 4. The interaction between SIRT6 and DDB2 was not mediated by DNA. HEK293 cells were transfected with vectors encoding pEGFP or DDB2-GFP and SIRT6-Flag. Cell lysates were immunoprecipitated with GFP-Trap in the presence of 50 mg/ml ethidium bromide (EtBr), followed by Western blotting.

Supplementary Figure 7. Related to Figure 4. SIRT6 interacts with DDB2 in A875 cells. **(A)** A875 cells were transfected with vectors encoding DDB2-GFP and SIRT6-Flag. At 24 h post transfection, cell lysates were immunoprecipitated with an anti-Flag antibody, followed by Western blotting. **(B)** A875 cells were transfected with vectors encoding pEGFP or DDB2-GFP and SIRT6-Flag. Cell lysates were immunoprecipitated with an anti-GFP antibody, followed by Western blotting.

Supplementary Figure 8. Related to Figure 4. PJ34 treatment did not abolish the stimulatory effect of SIRT6 on NER. HCA2-hTERT cells were pretreated with PJ34 at a concentration of 20 μM for 24 h before being transfected with 0.06 μg UVC-treated pmax-GFP and 15 ng pDsRed2-N1. After transfection, the cells were grown in complete medium supplemented with 20 μM PJ34 for 72 h before being harvested for FACS analysis. *** $P < 0.001$, ** $P < 0.01$.

Supplementary Figure 9. Related to Figure 4. SIRT6 regulates NER in a PKM2-independent manner. **(A)** Depleting PKM2 did not abolish the stimulatory effect of SIRT6 on NER. A control vector or vector encoding SIRT6 was cotransfected with 0.06 μg UVC-treated pmax-GFP together with 15 ng pDsRed2-N1 into control and PKM2-depleted HCA2-hTERT cells. At 72 h post transfection, the cells were harvested for FACS analysis. Error bars represent the s.d. ** $P < 0.01$, *$P < 0.05$. **(B)** Western blot analysis of PKM2 in control and PKM2-knockdown cells. **(C)** Depleting SIRT6 did not affect the interaction between DDB2 and DDB1. Control and SIRT6-depleted HEK293 cells were transfected with a control vector or vector encoding DDB2-GFP. At 24 h post transfection, cell lysates were immunoprecipitated with GFP-Trap, followed by Western blotting.

Supplementary Figure 10. Related to Figure 5. The interaction between the enzymatically dead mutant SIRT6 H133Y and DDB2 is not enhanced by UVC irradiation. HEK293 cells were cotransfected with SIRT6 H133Y-Flag and DDB2-GFP and treated with UVC (20 J/m^2) or left untreated. Then, the cells were harvested at the indicated time points. Cell lysates were immunoprecipitated with an anti-Flag antibody, followed by Western blot analysis with the indicated antibodies. IPed, immunoprecipitated.

Supplementary Figure 11. Related to Figure 5. DDB2 is acetylated by CBP and GCN5. HEK293 cells were cotransfected with plasmids encoding DDB2-EGFP-His and the acetyltransferase CBP or GCN5. At 24 h post transfection, the cells were harvested for immunoprecipitation with GFP-Trap, followed by Western blot analysis with the indicated antibodies.

Supplementary Figure 12. Related to Figure 5. DDB2 is not deacetylated by the enzymatically dead mutant SIRT6 H133Y. HEK293 cells were cotransfected with plasmids encoding DDB2-GFP and SIRT6 H133Y-Flag. At 16 h post transfection, the cells were harvested for immunoprecipitation with an antibody AcK, followed by Western blot analysis with the indicated antibodies.

Supplementary Figure 13. Related to Figure 5. The changes in the acetylation level of endogenous DDB2 in control and SIRT6-depleted HEK293 cells in response to UV irradiation. **(A)** UV irradiation led to a decrease in the acetylation level of DDB2 in HEK293 cells. HEK293 cells were irradiated with UVC (20 J/m^2) or left untreated, harvested and lysed. The cell lysates were immunoprecipitated with an antibody against AcK, followed by Western blot analysis with an antibody against DDB2. **(B)** In HEK293 cells with SIRT6 depletion, the UV irradiation-induced decline in the acetylation level of DDB2 was abolished. **(C)** In SIRT6-depleted HEK293 cells, the acetylation level was higher than that in control cells. The arrows indicate the acetylated endogenous DDB2. The asterisks indicate the heavy chains of the antibodies.

Supplementary Figure 14. Related to Figure 5. The interaction between the SIRT6 R65A mutant and DDB2 is not enhanced by UVC irradiation. HEK293 cells were cotransfected with SIRT6 R65A-Flag and DDB2-GFP and treated with UVC (20 J/m^2) or left untreated. Then, the cells were harvested at the indicated time points. Cell lysates were immunoprecipitated with an anti-Flag antibody, followed by Western blot analysis with the indicated antibodies. IPed, immunoprecipitated.

Supplementary Figure 15. Related to Figure 5. Identification of lysine residues that are potentially acetylated using mass spectrometry. **(A)** Three lysine residues in DDB2-GFP were potentially acetylated. DDB2-GFP was purified from HEK293FT cells prior to MS analysis. The MS/MS spectra of the peptides SPLELEPEAKK, TLHQHKLGR and ILQKAAPFDR from DDB2-GFP showed that K35, K40 and K77 were potentially acetylated. **(B)** K40 in DDB2 is not acetylated. DDB2-GFP WT and DDB2 K40R were transfected into HEK293 cells. GFP-tagged DDB2 was immunoprecipitated from cell lysates using an anti-acetylated lysine antibody and immunoblotted with the indicated antibodies. The arrow indicates the acetylated DDB2 WT or mutant.

Supplementary Figure 16. Related to Figure 5. The changes in the acetylation level of DDB2 2KR in control and SIRT6-depleted cells. Control and SIRT6-depleted HCA2-hTERT cells were transfected with a vector encoding DDB2-GFP 2KR. Then, the cells were harvested and lysed. Cell lysates were immunoprecipitated with GFP-Trap, followed by Western blot analysis with the indicated antibodies.

Supplementary Figure 17. Related to Figure 6. SIRT6 does not affect DDB2 protein levels. **(A)** Western blot analysis of the expression level of DDB2 in SIRT6-depleted cells. **(B)** Western blot analysis of the expression level of DDB2 in control and SIRT6-overexpressing cells.

Supplementary Figure 18. Related to Figure 6. Comparison of DDB2 ubiquitination levels between cells overexpressing SIRT6 WT and those overexpressing the enzymatically dead mutant SIRT6 H133Y. HEK293 cells were transfected with a DDB2-Flag plasmid and vectors encoding either SIRT6 WT or SIRT6 H133Y. After 16 h, the cells were harvested for immunoprecipitation with an antibody against Flag, followed by Western blot analysis with an antibody recognizing ubiquitin.

Supplementary Figure 19. Related to Figure 7. SIRT6 mutants partially abolished the stimulatory effect on NER. **(A)** Several SIRT6 mutants lost the ability to enhance NER efficiency in A875 cells. *** $P < 0.001$, ** $P < 0.01$. **(B)** The SIRT6 P27S and H50Y mutants partially lost their deacetylase activity specific for H3K56Ac. HCA2-hTERT cells were transfected with 5 µg SIRT6 WT or mutants. At 16 h post transfection, the cells were harvested for Western blot analysis with the indicated antibodies. **(C)** SIRT6 mutants failed to promote the removal of DDB2 from chromatin in response to UV irradiation. SIRT6-depleted HCA2-hTERT cells were transfected with a control vector or vectors expressing SIRT6 WT or SIRT6 mutants and then irradiated with UVC (20 J/m^2). The irradiated cells were harvested at 5 min post UV irradiation and subjected to cellular protein fractionation, followed by Western blot analysis for DDB2 in the chromatin fractions. ImageJ software was used for quantification.

Supplementary Figure 20. The model for SIRT6 promoting NER by deacetylating DDB2. Upon UV irradiation, the UV-DDB complex translocates onto the damaged chromatin by binding to lesions and facilitates the binding of XPC. In response to UV exposure, SIRT6 is recruited to DNA damage sites, where it interacts with and deacetylates DDB2, promoting DDB2 polyubiquitination. Then, the chromatin-associated ubiquitinated DDB2 interacts with the segregase p97 and is removed from the chromatin, facilitating the NER signaling cascade.

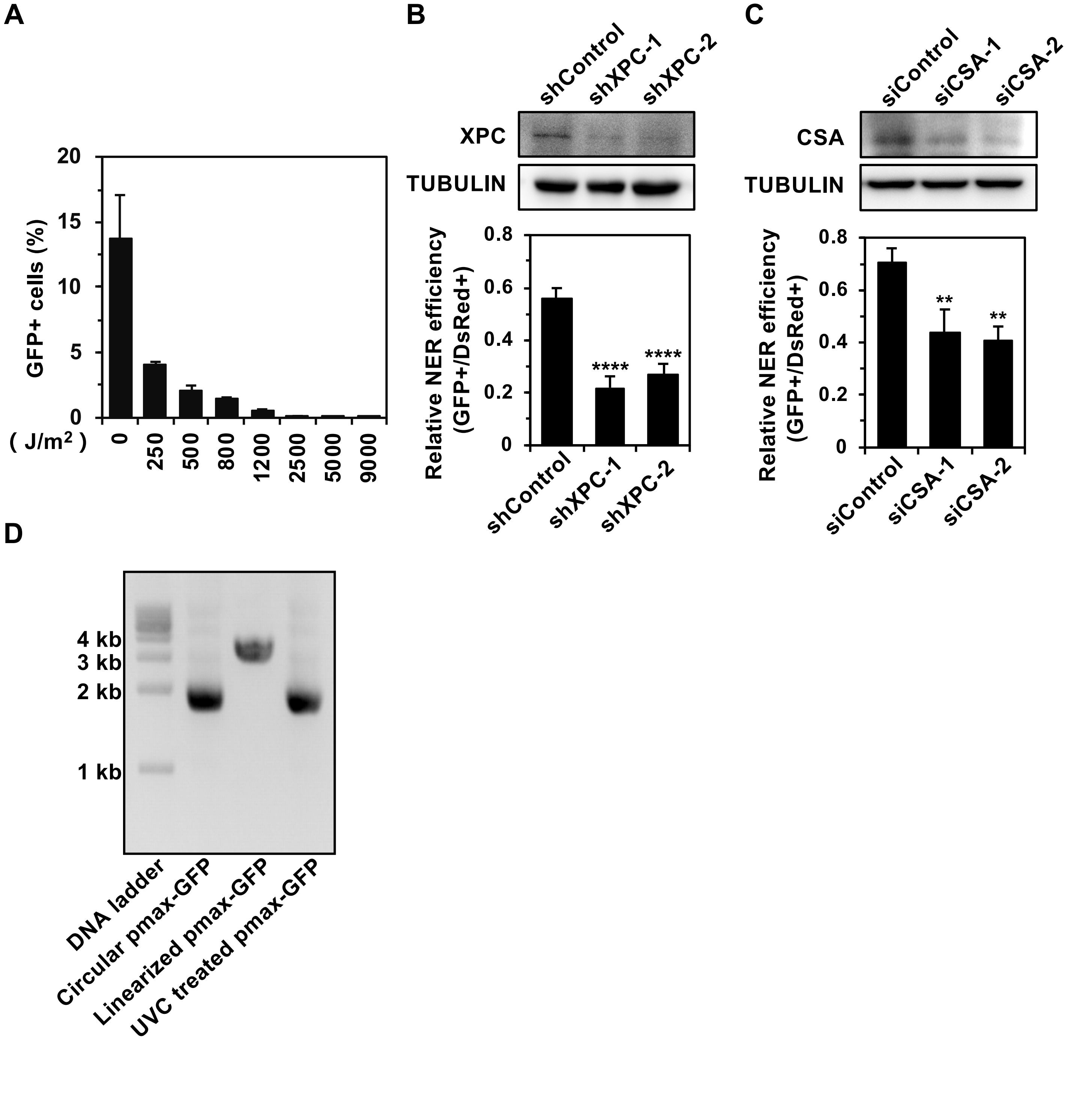
A
B
C
D
GFP+ cells (%)
20
15
10
5
0
(J/m2)
0
250
500
800
1200
2500
5000
9000
shControl
shXPC-1
shXPC-2
XPC
TUBULIN
Relative NER efficiency (GFP+/DsRed+)
0.8
0.6
0.4
0.2
0
****
****
siControl
siCSA-1
siCSA-2
CSA
TUBULIN
**
**
4 kb
3 kb
2 kb
1 kb
DNA ladder
Circular pmax-GFP
Linearized pmax-GFP
UVC treated pmax-GFP

Figure S1

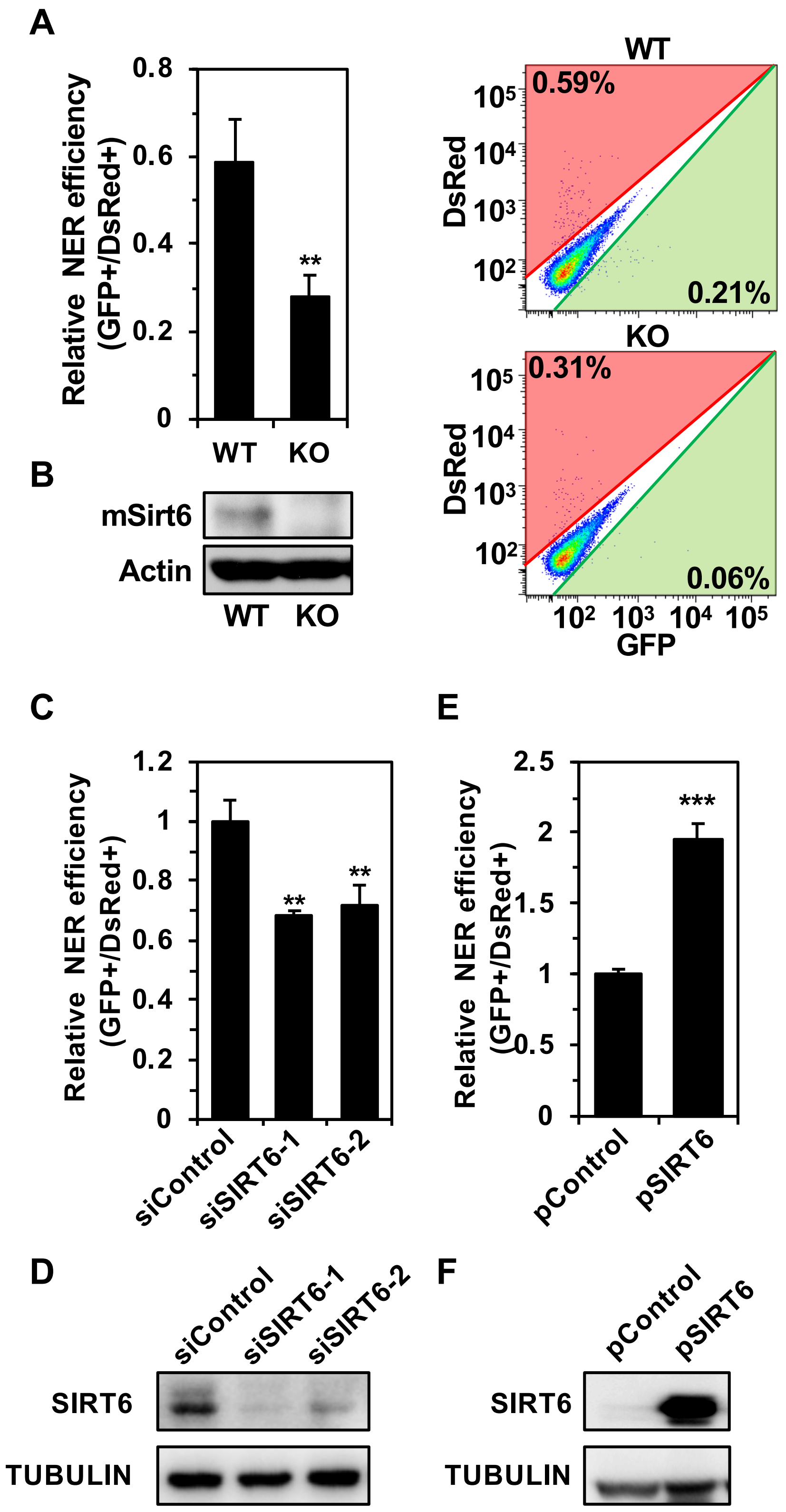
A
Relative NER efficiency (GFP+/DsRed+)
0.8
0.6
0.4
0.2
0
WT
KO
**
WT
KO
DsRed
GFP
0.59%
0.21%
0.31%
0.06%
B
mSirt6
Actin
WT
KO
C
Relative NER efficiency (GFP+/DsRed+)
1.2
1
0.8
0.6
0.4
0.2
0
siControl
siSIRT6-1
siSIRT6-2
**
**
E
Relative NER efficiency (GFP+/DsRed+)
2.5
2
1.5
1
0.5
0
pControl
pSIRT6
***
D
siControl
siSIRT6-1
siSIRT6-2
SIRT6
TUBULIN
F
pControl
pSIRT6
SIRT6
TUBULIN

Figure S2

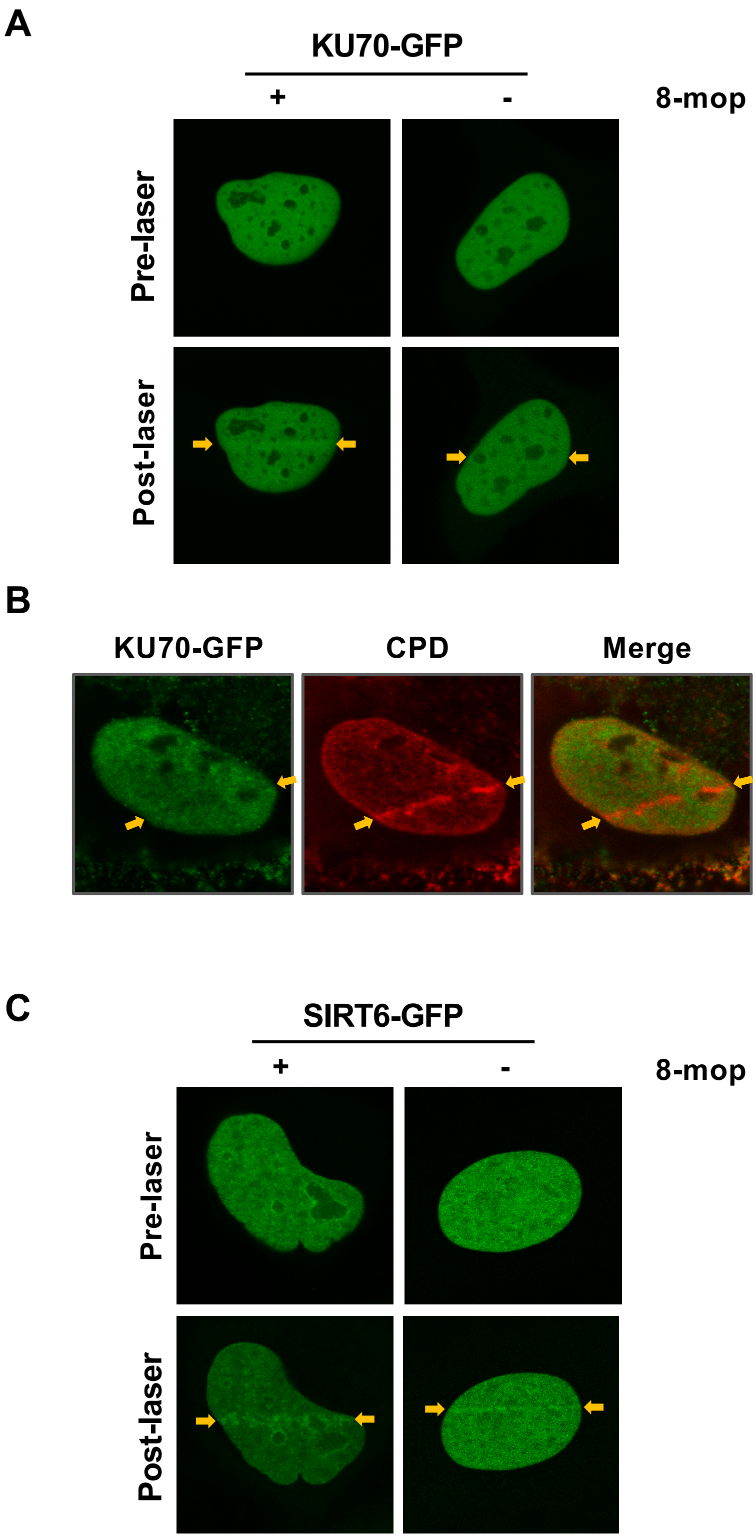
A
KU70-GFP
+
-
8-mop
Pre-laser
Post-laser
B
KU70-GFP
CPD
Merge
C
SIRT6-GFP
+
-
8-mop
Pre-laser
Post-laser

Figure S3

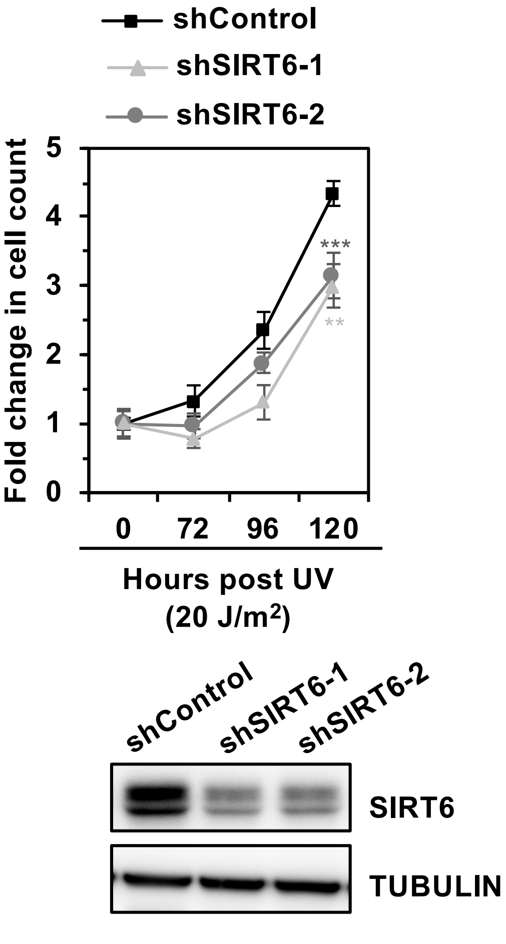
shControl
shSIRT6-1
shSIRT6-2
Fold change in cell count
0
1
2
3
4
5
***
**
0
72
96
120
Hours post UV
(20 J/m2)
shControl
shSIRT6-1
shSIRT6-2
SIRT6
TUBULIN

Figure S4

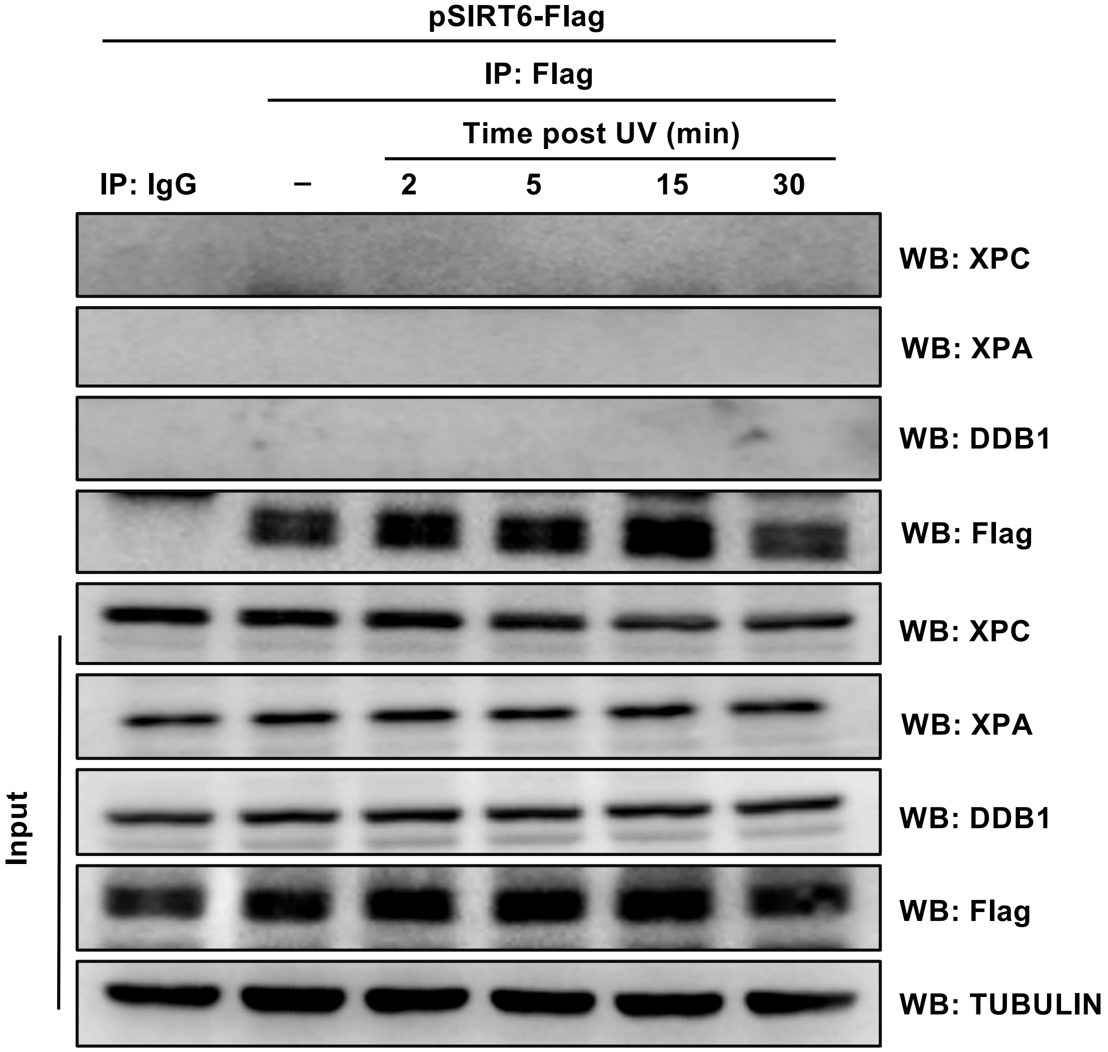
pSIRT6-Flag
IP: Flag
Time post UV (min)
IP: IgG
–
2
5
15
30
WB: XPC
WB: XPA
WB: DDB1
WB: Flag
WB: XPC
WB: XPA
WB: DDB1
WB: Flag
WB: TUBULIN
Input

Figure S5

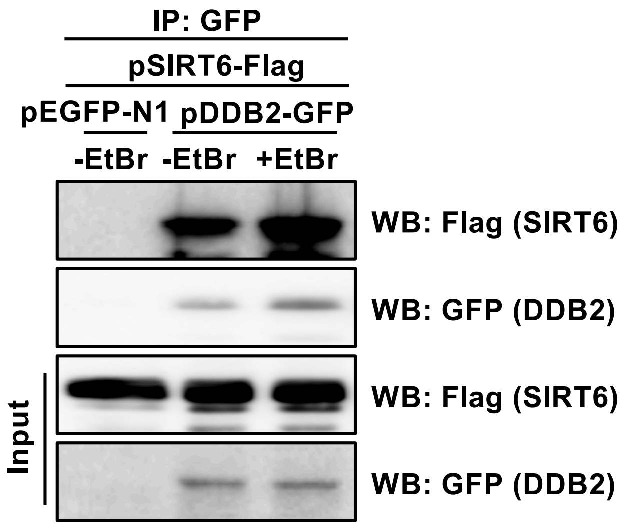
IP: GFP
pSIRT6-Flag
pEGFP-N1
pDDB2-GFP
-EtBr
-EtBr
+EtBr
WB: Flag (SIRT6)
WB: GFP (DDB2)
Input
WB: Flag (SIRT6)
WB: GFP (DDB2)

Figure S6

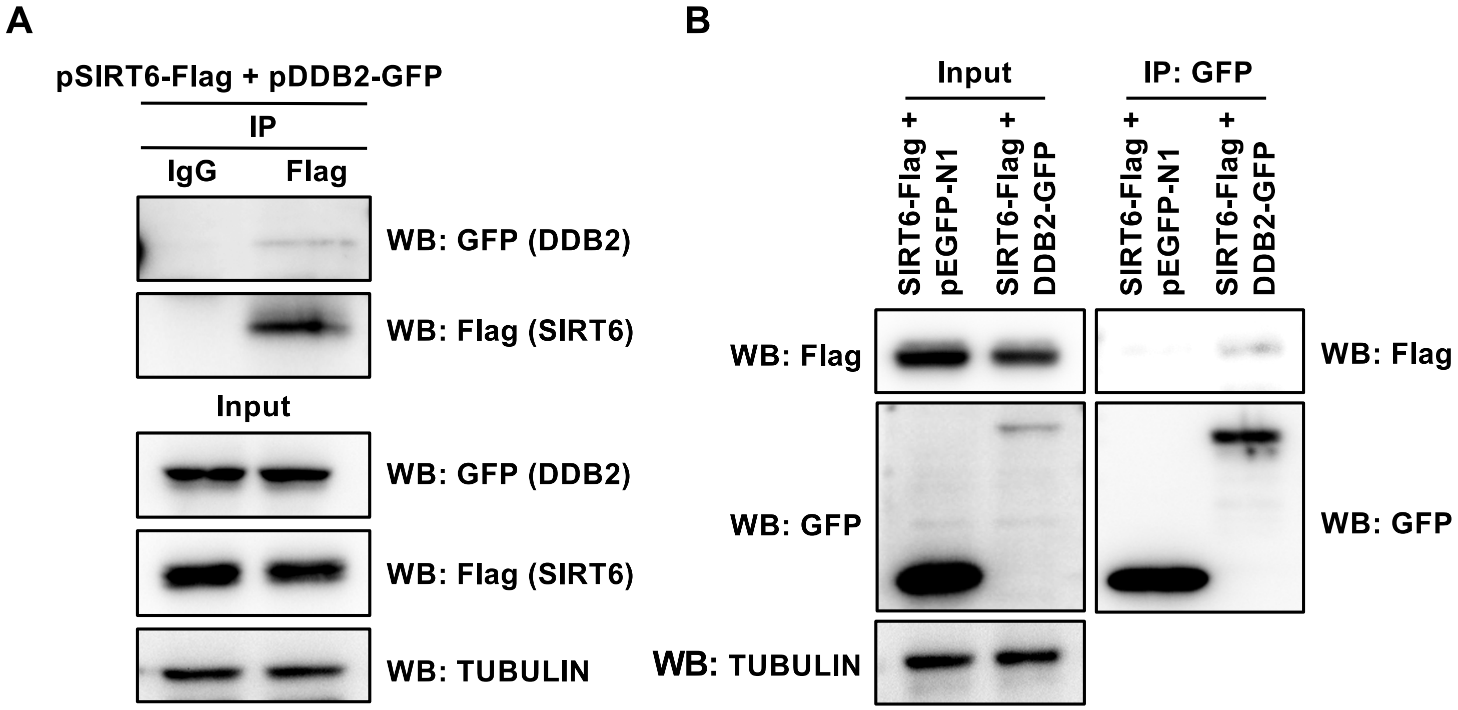

A
pSIRT6-Flag + pDDB2-GFP
IP
IgG
Flag
WB: GFP (DDB2)
WB: Flag (SIRT6)
Input
WB: GFP (DDB2)
WB: Flag (SIRT6)
WB: TUBULIN
B
Input
IP: GFP
SIRT6-Flag + pEGFP-N1
SIRT6-Flag + DDB2-GFP
SIRT6-Flag + pEGFP-N1
SIRT6-Flag + DDB2-GFP
WB: Flag
WB: Flag
WB: GFP
WB: GFP
WB: TUBULIN

Figure S7

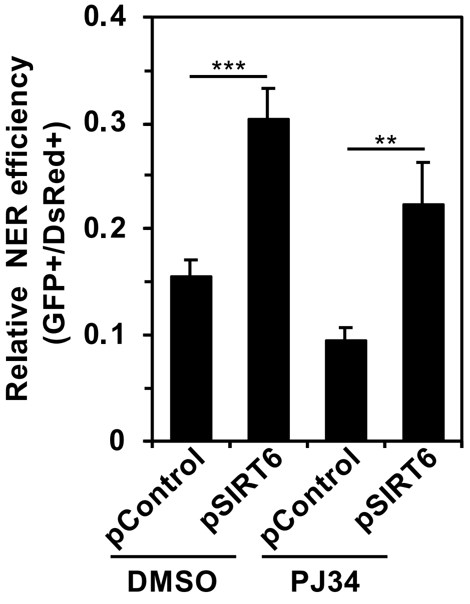
0.4
0.3
0.2
0.1
0
Relative NER efficiency
(GFP+/DsRed+)
***
**
pControl
pSIRT6
pControl
pSIRT6
DMSO
PJ34

Figure S8

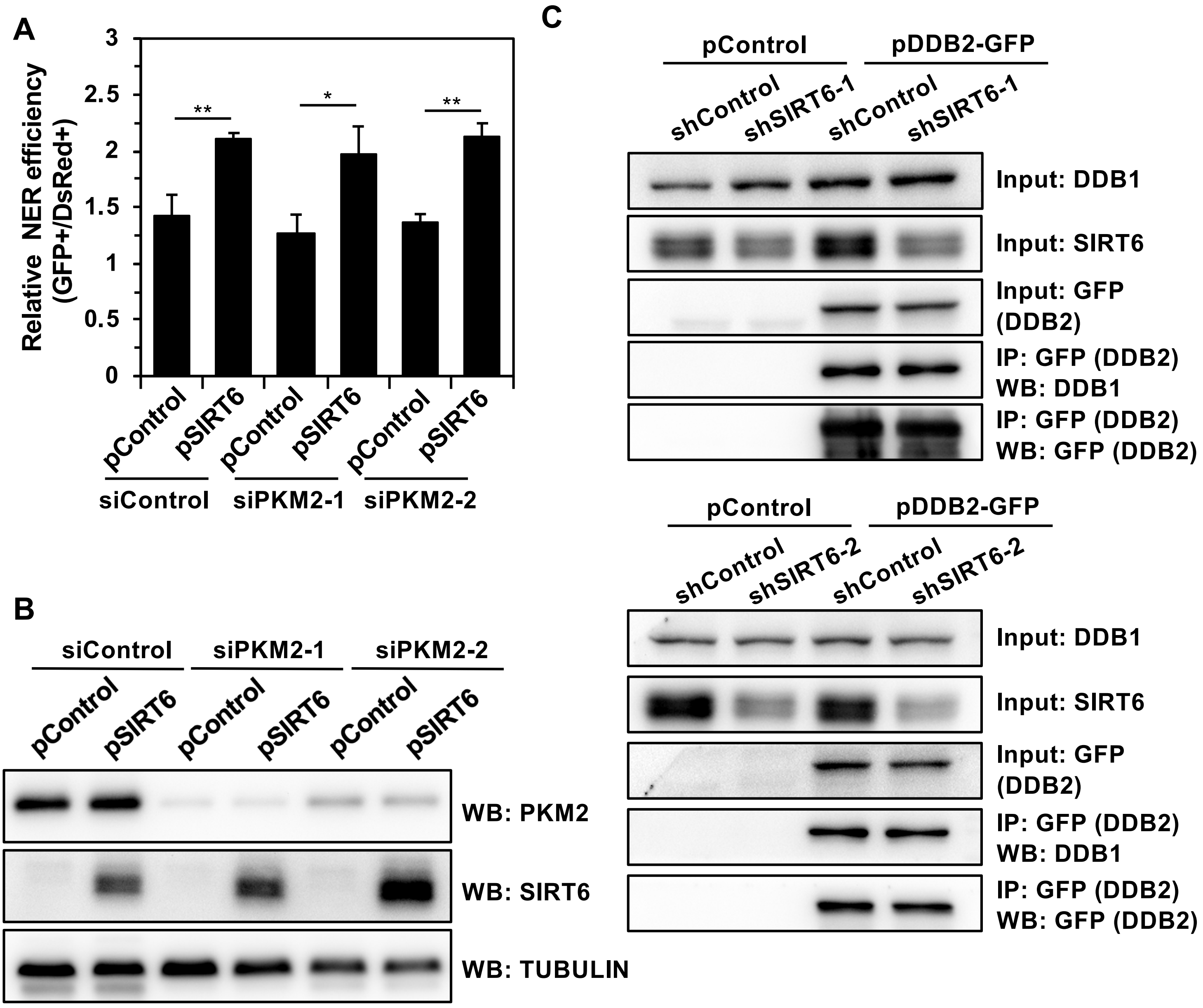
A
Relative NER efficiency (GFP+/DsRed+)
3
2.5
2
1.5
1
0.5
0
**
*
**
pControl
pSIRT6
pControl
pSIRT6
pControl
pSIRT6
siControl
siPKM2-1
siPKM2-2
B
siControl
siPKM2-1
siPKM2-2
pControl
pSIRT6
pControl
pSIRT6
pControl
pSIRT6
WB: PKM2
WB: SIRT6
WB: TUBULIN
C
pControl
pDDB2-GFP
shControl
shSIRT6-1
shControl
shSIRT6-1
Input: DDB1
Input: SIRT6
Input: GFP (DDB2)
IP: GFP (DDB2) WB: DDB1
IP: GFP (DDB2) WB: GFP (DDB2)
pControl
pDDB2-GFP
shControl
shSIRT6-2
shControl
shSIRT6-2
Input: DDB1
Input: SIRT6
Input: GFP (DDB2)
IP: GFP (DDB2) WB: DDB1
IP: GFP (DDB2) WB: GFP (DDB2)

Figure S9

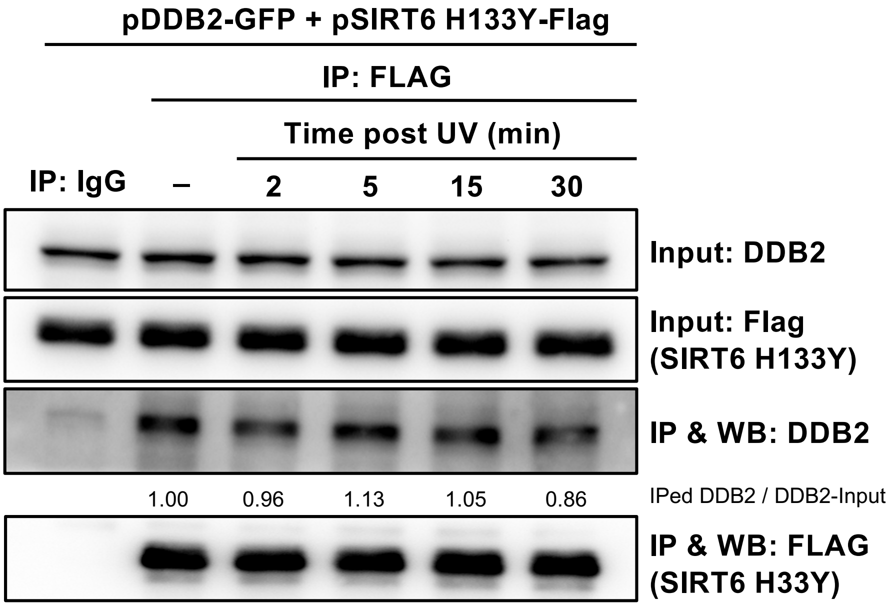
pDDB2-GFP + pSIRT6 H133Y-Flag
IP: FLAG
Time post UV (min)
IP: IgG
–
2
5
15
30
Input: DDB2
Input: Flag (SIRT6 H133Y)
IP & WB: DDB2
1.00
0.96
1.13
1.05
0.86
IPed DDB2 / DDB2-Input
IP & WB: FLAG (SIRT6 H33Y)

Figure S10

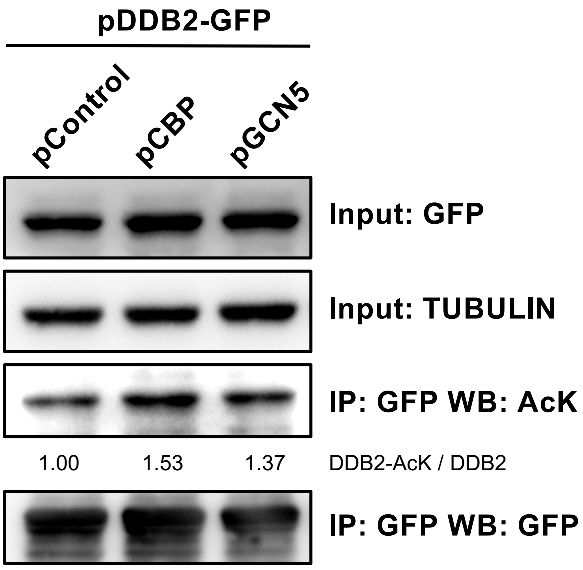
pDDB2-GFP
pControl
pCBP
pGCN5
Input: GFP
Input: TUBULIN
IP: GFP WB: AcK
1.00
1.53
1.37
DDB2-AcK / DDB2
IP: GFP WB: GFP

Figure S11

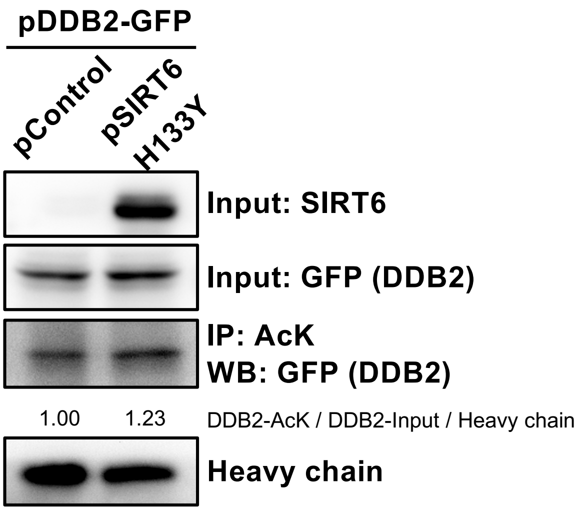
pDDB2-GFP
pControl
pSIRT6 H133Y
Input: SIRT6
Input: GFP (DDB2)
IP: AcK
WB: GFP (DDB2)
1.00
1.23
DDB2-AcK / DDB2-Input / Heavy chain
Heavy chain

Figure S12

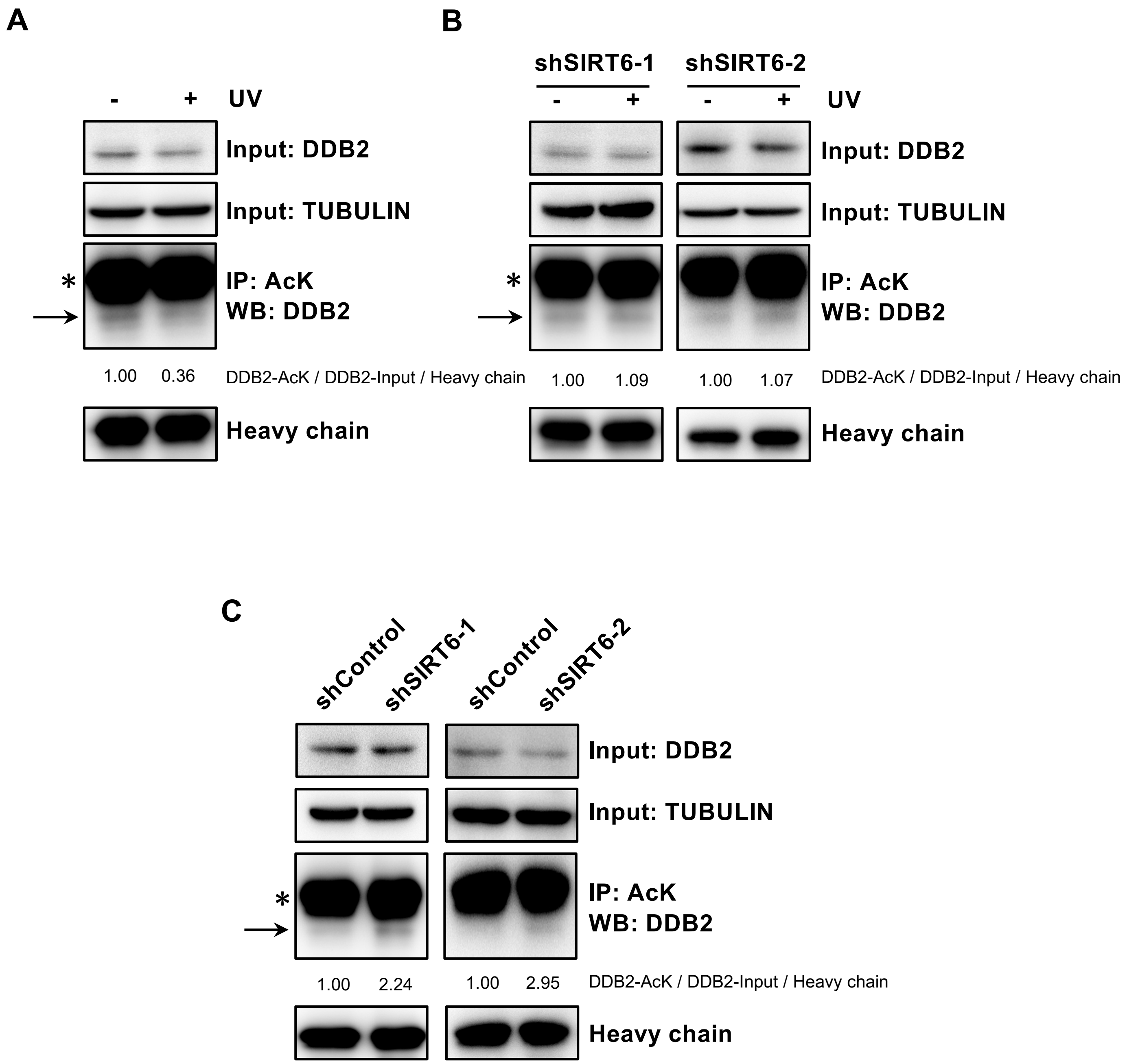
A
- + UV
Input: DDB2
Input: TUBULIN
IP: AcK
WB: DDB2
1.00 0.36
DDB2-AcK / DDB2-Input / Heavy chain
Heavy chain
B
shSIRT6-1
shSIRT6-2
- + - + UV
Input: DDB2
Input: TUBULIN
IP: AcK
WB: DDB2
1.00 1.09 1.00 1.07
DDB2-AcK / DDB2-Input / Heavy chain
Heavy chain
C
shControl
shSIRT6-1
shControl
shSIRT6-2
Input: DDB2
Input: TUBULIN
IP: AcK
WB: DDB2
1.00 2.24 1.00 2.95
DDB2-AcK / DDB2-Input / Heavy chain
Heavy chain

Figure S13

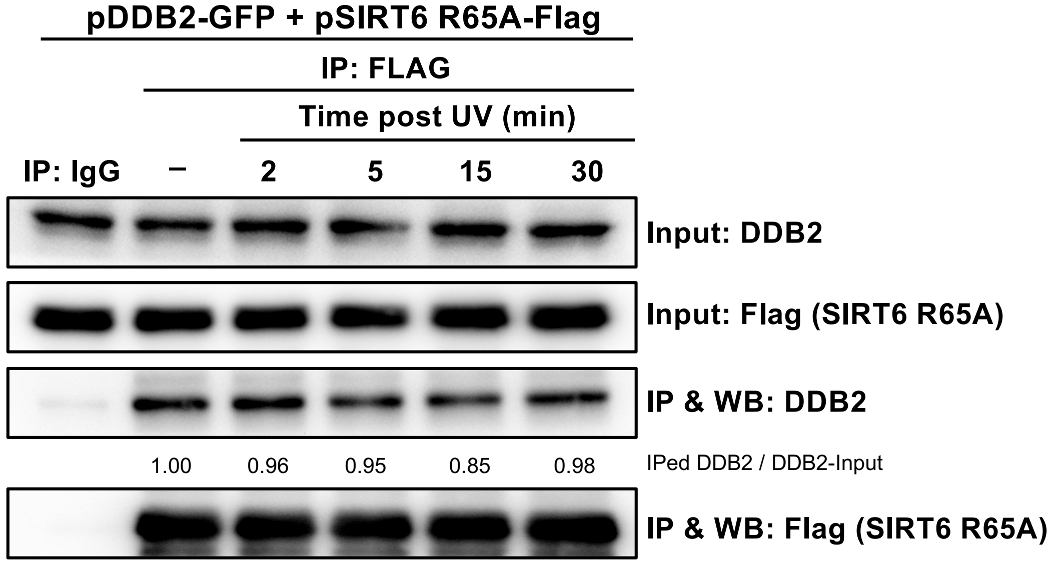
pDDB2-GFP + pSIRT6 R65A-Flag
IP: FLAG
Time post UV (min)
IP: IgG
–
2
5
15
30
Input: DDB2
Input: Flag (SIRT6 R65A)
IP & WB: DDB2
1.00
0.96
0.95
0.85
0.98
IPed DDB2 / DDB2-Input
IP & WB: Flag (SIRT6 R65A)

Figure S14

A

Sequence: SPLELEPEAKK, K10-Acetyl (42.01057 Da)

Charge: +2,   Monoisotopic m/z: 641.84741 Da

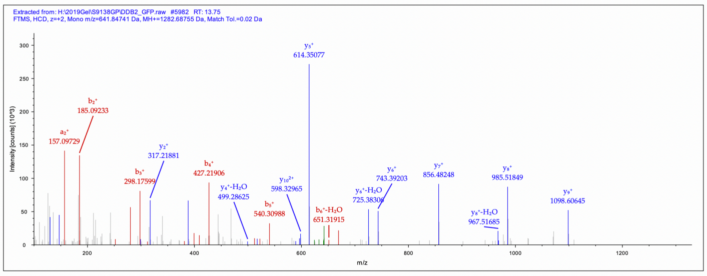

Sequence: TLHQHKLGR, K6-Acetyl (42.01057 Da)

Charge: +2,   Monoisotopic m/z: 566.32294 Da

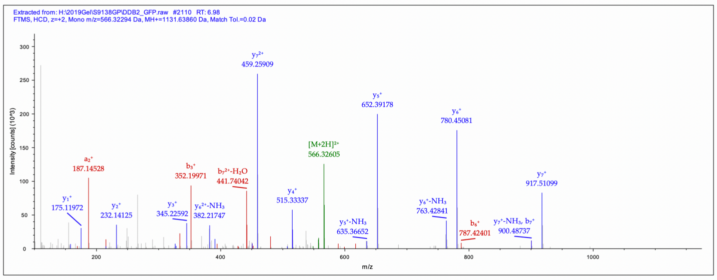

Sequence: ILQKAAPFDR, K4-Acetyl (42.01057 Da)

Charge: +2,   Monoisotopic m/z: 600.83997 Da

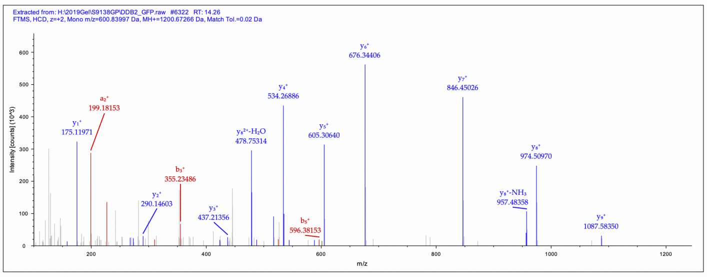

B

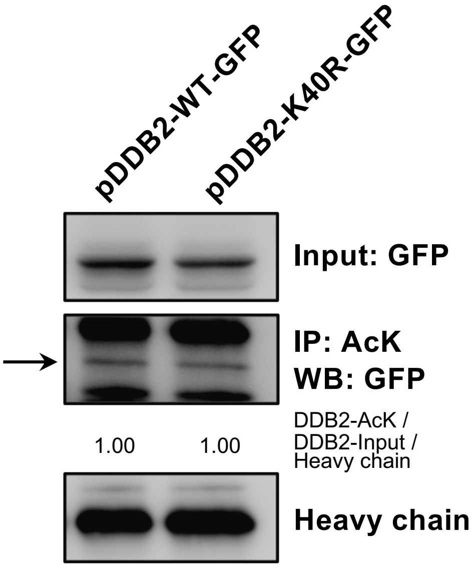

Figure S15

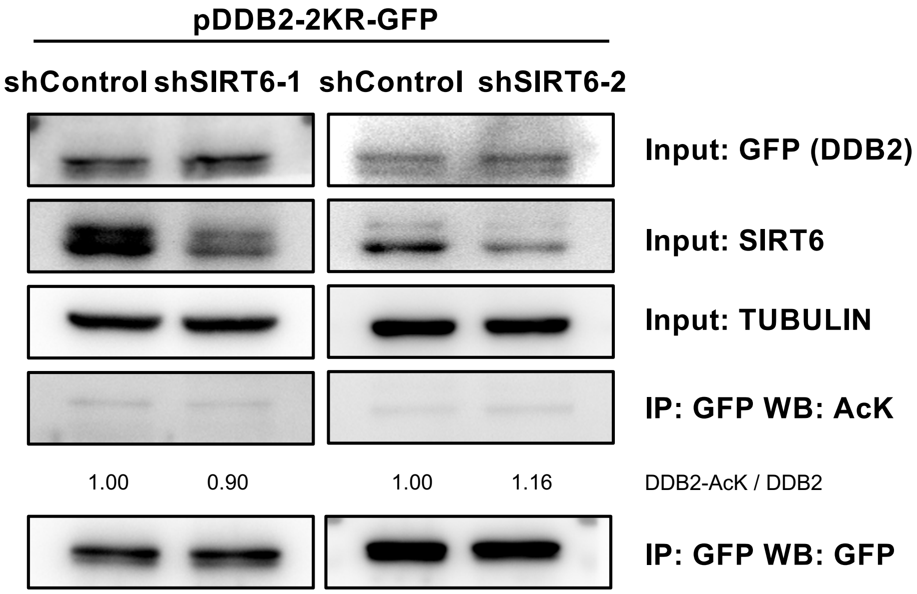
pDDB2-2KR-GFP
shControl shSIRT6-1 shControl shSIRT6-2
Input: GFP (DDB2)
Input: SIRT6
Input: TUBULIN
IP: GFP WB: AcK
1.00 0.90 1.00 1.16
DDB2-AcK / DDB2
IP: GFP WB: GFP

Figure S16

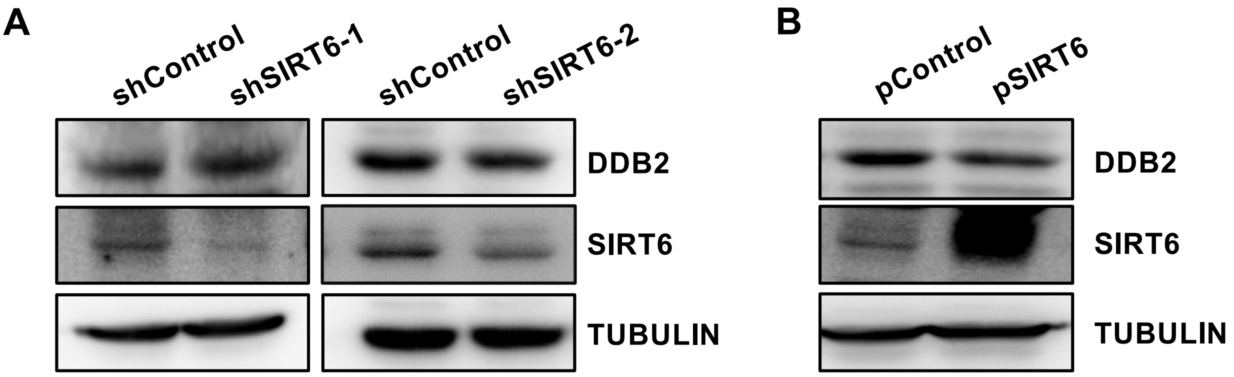
A
shControl
shSIRT6-1
shControl
shSIRT6-2
DDB2
SIRT6
TUBULIN
B
pControl
pSIRT6
DDB2
SIRT6
TUBULIN

Figure S17

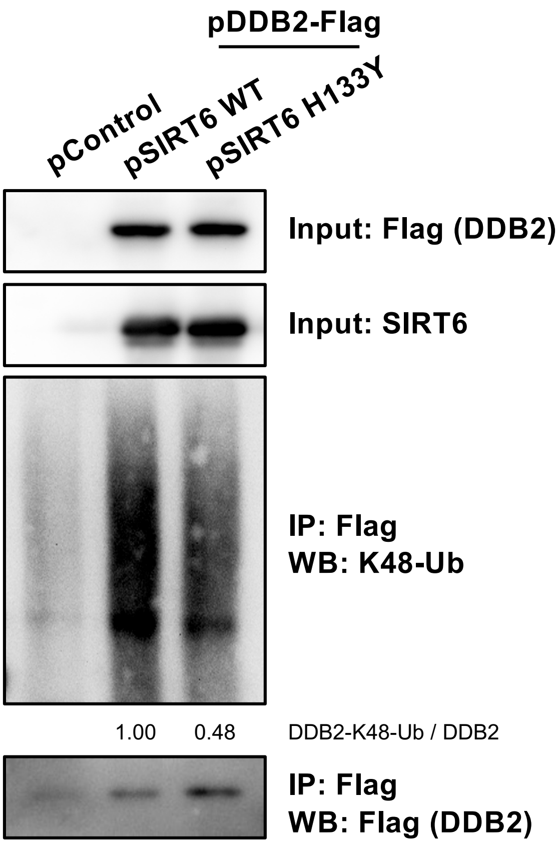
pDDB2-Flag
pControl
pSIRT6 WT
pSIRT6 H133Y
Input: Flag (DDB2)
Input: SIRT6
IP: Flag
WB: K48-Ub
1.00
0.48
DDB2-K48-Ub / DDB2
IP: Flag
WB: Flag (DDB2)

Figure S18

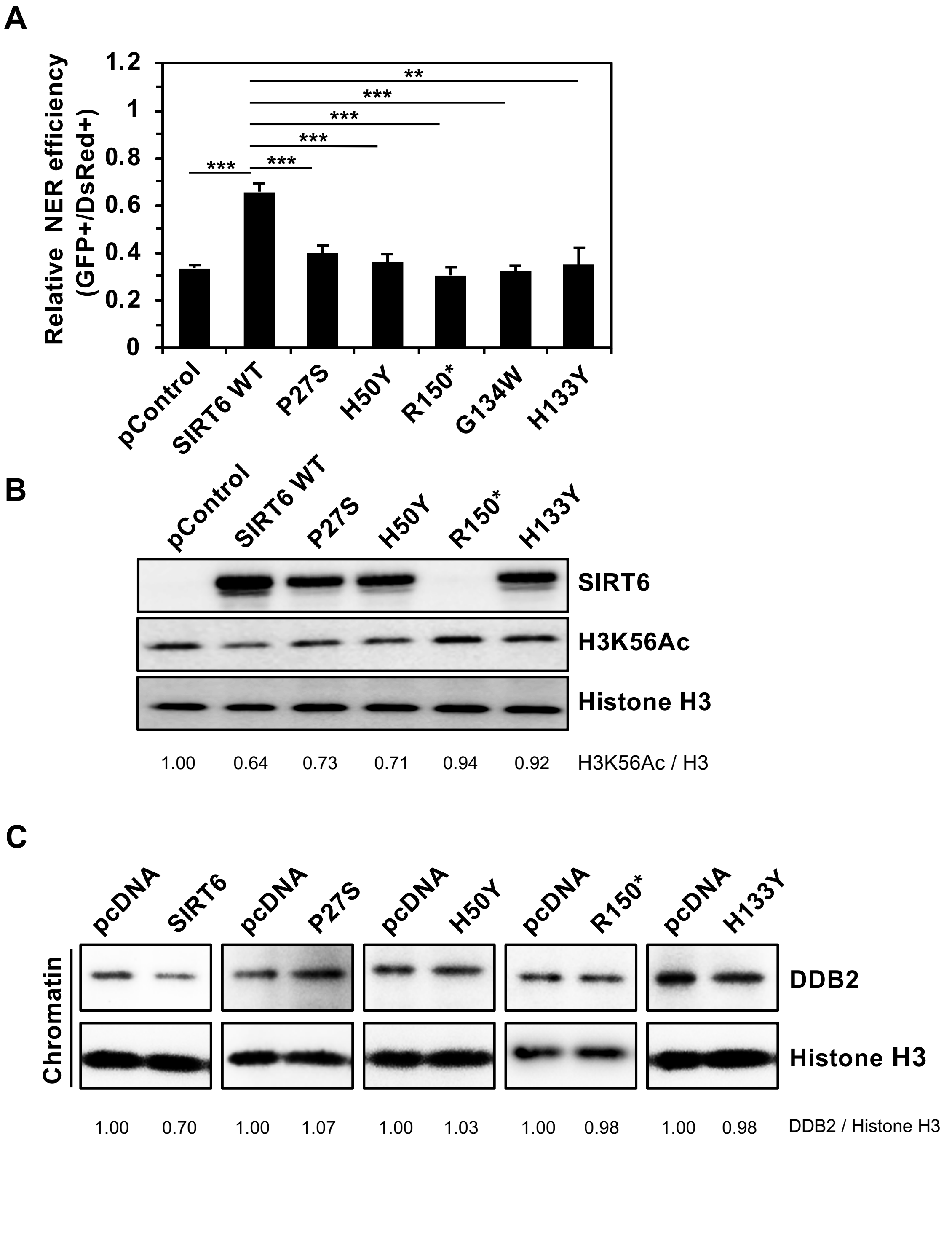
A
Relative NER efficiency (GFP+/DsRed+)
1.2
1
0.8
0.6
0.4
0.2
0
**
***
***
***
***
***
pControl
SIRT6 WT
P27S
H50Y
R150*
G134W
H133Y
B
pControl
SIRT6 WT
P27S
H50Y
R150*
H133Y
SIRT6
H3K56Ac
Histone H3
1.00
0.64
0.73
0.71
0.94
0.92
H3K56Ac / H3
C
pcDNA
SIRT6
pcDNA
P27S
pcDNA
H50Y
pcDNA
R150*
pcDNA
H133Y
Chromatin
DDB2
Histone H3
1.00
0.70
1.00
1.07
1.00
1.03
1.00
0.98
1.00
0.98
DDB2 / Histone H3

Figure S19

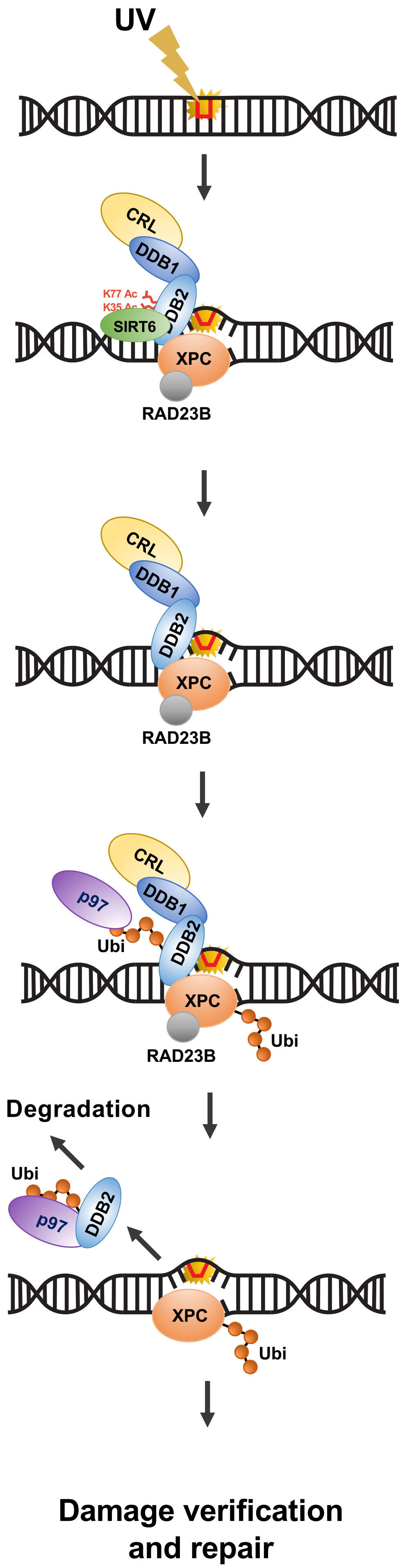
UV
CRL
DDB1
K77 Ac
SIRT6
DDB2
XPC
RAD23B
p97
Ubi
Degradation
Damage verification
and repair

Figure S20
